# Multiple Reputations: Selective Attention to Competence and Character

**DOI:** 10.1177/01461672241301116

**Published:** 2024-12-06

**Authors:** Alexandra V. T. de La Trobe, Gordon D. A. Brown, Lukasz Walasek

**Affiliations:** 1 University of Warwick, Coventry, UK

**Keywords:** reputation, helpfulness, competence, person perception, investment game

## Abstract

Reputation is multidimensional, with some traits being more relevant than others in particular contexts. Can people selectively respond to reputational cues relevant to the task at hand? Across three studies, we examined how people weigh cues about helpfulness and competence when forming expectations about strangers’ behavior. Using adapted investment games, we varied whether a stranger’s helpfulness or competence predicted participants’ future payoffs. We found that when helpfulness is task-relevant (Experiments 1 and 2), participants correctly use this cue in investment decisions. When competence matters most (Experiment 3), participants use it as the primary cue. Overall, a high reputation for outcome-irrelevant characteristics did not compensate for a low reputation for the outcome-relevant reputational cue. However, we also find an asymmetric spillover: Decision-makers prefer cooperating with others who are highly competent and highly helpful, regardless of task demands. We discuss our results within the theoretical framework of person perception and theories of reputation.

## Introduction

As humans, we frequently find ourselves in situations where we must choose whether and how much to cooperate with others. However, this brings the need to decide which people are the most suitable partners. Would you be willing to invest in a joint project with someone you have just met? If you do invest, how would you estimate the probability of the stranger being competent and trustworthy? In many everyday interactions, such judgments must be informed by our knowledge of others’ reputations when we have not previously interacted with the relevant individual (e.g., Dores Cruz, Thielmann, et al., 2021; Giardini et al., 2022). Our ability to learn about a person’s character and abilities from their past behavior with others—through their reputation—is therefore an essential underpinning of indirect reciprocity (Berg et al., 1995; Brandt et al., 2003; Brandt & Sigmund, 2004; Nowak, 2006; Nowak & Sigmund, 1998; Ohtsuki & Iwasa, 2004, 2006; Seinen & Schram, 2006; van Dijk & De Dreu, 2021). Reputations allow us to form expectations about an individual’s future behavior and decisions toward us (André, 2010; Barclay, 2013, 2016; Dores Cruz, Thielmann, et al., 2021; Yao et al., 2014) and, therefore, play a crucial role in bridging the information asymmetries that are common in many social interactions (Akerlof, 1970; Arrow, 1963; Bolton et al., 2005; Nowak & Sigmund, 1998; Spence, 1974).

Yet, in many everyday social contexts, the task of forming an impression about a stranger is difficult due to the multidimensional nature of reputations. Reputation reflects multiple characteristics related to a person’s skills and abilities, as well as their personality and values (Barclay, 2013, 2015, 2016). A person who has a reputation of being trustworthy may not be competent, whereas a highly skilled individual may be known for being immoral. Furthermore, not all aspects of one’s reputation are relevant in all situations. Depending on the context, a person’s reputation on one character trait may be more informative than another (Barclay, 2015; Garfield et al., 2021; Giardini et al., 2022; Romano et al., 2021). In some situations, we might be concerned about the ability of a potential co-worker to do well on a task that requires mastery of a specific skill (e.g., hunting or farming) (Apicella, 2014; Apicella et al., 2012; Gurven et al., 2000; Macfarlan & Lyle, 2015; K. M. Smith & Apicella, 2020; K. M. Smith et al., 2022), but in other contexts, we might worry whether a potential new business partner will abscond with the company’s cash. The central question of this paper is whether and to what extent people are capable of adaptively choosing whom to work with on the basis of the most task-relevant aspects of their reputation. In a series of experiments with real monetary incentives, we tested whether decision-makers can prioritize and selectively consider only the aspects of one’s reputation that matter for their chances of maximizing their personal gain.

The remainder of the paper is structured as follows. We begin by briefly summarizing research on the role of reputation in social interactions before we turn to a discussion of the key trait-based frameworks of reputation. We draw a parallel between the research in evolutionary and social psychology to define key dimensions of reputation that are a focus of this paper, namely warmth/morality and competence. Next, we review the evidence demonstrating how reputations on various traits inform people’s decisions about whom to interact and cooperate with. This brings us to the focus of this work, which concerns situations when decision-makers must prioritize one cue about one’s reputation over another to identify the best partner for a cooperative task.

### Approaches to Reputation

Evolutionary psychologists have examined the role of reputation by viewing social interactions through the lens of *biological markets* (Barclay, 2013, 2016; Noë & Hammerstein, 1994, 1995). The dynamics of social and romantic cooperative interactions, such as selecting friends or reproductive partners, are taken to resemble a competitive market, where individuals choose whom to cooperate with to achieve specific tasks or goals (Barclay, 2013). There is ample evidence that individuals tend to—consciously or unconsciously—choose partners who can offer the relatively highest quality compared to everyone else (Barclay, 2013, 2016; Hayashi & Yamagishi, 1998; Noë & Hammerstein, 1995).

Since cooperation opportunities are limited and consequently not everyone can benefit from cooperating, individuals compete to be chosen by signaling their good reputation (Barclay, 2013, 2016; Noë & Hammerstein, 1994, 1995). To increase their chances of being selected as a partner, people (and non-human animals) therefore signal desirable traits in an attempt to build a reputation as an attractive partner (Barclay, 2013, 2016; Barclay & Van Vugt, 2015; McNamara et al., 2008). When signaling a particular trait is especially costly—requiring significant time, effort, or resources—such signaling can positively affect an individual’s reputation (Grafen, 1990; Zahavi, 1977; Zahavi & Zahavi, 1997), as high-cost signals are relatively less costly for high-quality individuals compared with low-quality individuals (Barclay, 2016).

Beyond signaling our own best qualities, people must also be able to accurately learn about the reputations of other people. How do we decide who might be a good cooperative partner, and whom to avoid? Models of impression formation and person perception are rooted in the evolutionary pressures that made it essential for people to distinguish “friend from foe” (Fiske et al., 2007). The dominant view is that when making judgments about others, people initially assess their intentions and then evaluate their ability to act on them (Abele & Wojciszke, 2007; Cuddy et al., 2008; Fiske et al., 2007; Judd et al., 2005; Peeters, 2008; Peters & Kashima, 2015; Rosenberg et al., 1968; Wojciszke, 2005). Perceptions of reputation play a central role in our understanding of how people navigate various social situations and, therefore, encompass a wide range of topics in the field of social cognition, including stereotype formation, perception of status, social categorization, attribution theory, and implicit biases, among others (Mattan et al., 2017). For example, one well-studied mechanism by which people learn about the reputation of others is via *gossip* (e.g., Brambilla et al., 2011, 2021; Dores Cruz, Nieper, et al., 2021; Giardini et al., 2022; Goodwin, 2015; Goodwin et al., 2014; Hackel et al., 2015, 2022; Hess & Hagen, 2021). In the absence of direct experiences, individuals tend to rely on gossip to form reputations and decide how cooperatively to behave toward a stranger (Baum et al., 2020; Dores Cruz, Thielmann, et al., 2021; Emler, 2019; Fonseca & Peters, 2018; Giardini et al., 2022; Hauke & Abele, 2020; Hess & Hagen, 2021; Mesoudi et al., 2006; Peters & Kashima, 2015). Gossip can also be used strategically, such as when one person uses it to elevate (harm) the target’s reputation, with the goal of increasing (diminishing) the target’s potential gain from future interactions and cooperation with others (Dores Cruz, Nieper, et al., 2021; Giardini et al., 2022; Hess & Hagen, 2021; Milinski, 2019; Molho et al., 2020; Sommerfeld et al., 2007; Wu et al., 2016).

Taken together, an account of how reputations are signaled and perceived is essential for understanding many social interactions. However, to fully appreciate the role of reputation in everyday decision-making, it is important to recognize its multidimensional and context-dependent nature.

### Dimensions of Reputation

It is widely recognized that reputation is not a unitary construct (Barclay, 2013, 2015, 2016). An individual’s reputation is a set of subjective beliefs that others hold about their character and abilities (Barclay, 2013, 2015; Giardini et al., 2022; Molho et al., 2020). Although many unique dimensions have been identified across different research streams, and varied terminology has been used to describe reputations, parallels can again be drawn between evolutionary and social psychology research (Barclay, 2013; Brambilla et al., 2021).

Research on biological markets for human cooperative behavior in evolutionary psychology recognizes the importance of multiple traits, not just reciprocity. The market value of a potential partner may, therefore, be determined by their *qualities*, *tendencies*, and *availability* (Barclay, 2013; Kummer, 1978; Tooby & Cosmides, 1996). Thus, in deciding on the suitability of a potential cooperation partner, a decision-maker may rely on cues that signal their physical and intellectual prowess as well as their readiness/willingness to cooperate. Between these two important indicators of partner selection, reputation traits can correspond to physical, dispositional, and social characteristics (Barclay, 2015; Barclay & Reeve, 2012). For example, an individual may engage in social signaling of their intelligence by achieving higher qualifications, of their physical strength by participating in competitions, or of their social connections by affiliating with groups and high-status individuals (Barclay, 2016). Similarly, people may signal their willingness to help others by donating, volunteering, or being more attentive to the needs and demands made by others (André, 2010; Ohtsubo & Watanabe, 2009; E. A. Smith & Bird, 2005).

Insights about the core dimensions of reputation come from studies of social perception (of both groups and individuals) in social cognition (for reviews, see Fiske et al., 2007; Koch et al., 2021). Early work defined these two dimensions as *warmth* (intentions to harm or help) and *competence* (ability to act on these intentions) (Chen & Guo, 2021; Fiske, 1980, 1993; Fiske et al., 2007; Wojciszke et al., 1998). According to the original two-factor model proposed by Fiske and colleagues, the warmth dimension includes traits such as trustworthiness, cooperativeness, and sincerity, while competence encompasses qualities such as efficiency, competence, and capability (Fiske, 1980, 1993; Fiske et al., 2007; Wojciszke, 1994). Two dimensions have also been labeled as quality and intention (Roberts, 2015), communion and agency (Abele, 2022; Abele & Wojciszke, 2007, 2014; Brambilla & Leach, 2014; Wojciszke & Abele, 2018), capital and character (Barker et al., 2019), and morality and competence (Abele et al., 2008; Brambilla et al., 2021; Fiske, 2018; Fiske et al., 2007; Wojciszke, 1994, 2005). One particularly notable development in social perception literature is the distinction between *warmth* and *morality* dimensions. Although the labels *warmth* and *morality* have often been used interchangeably in the past (Abele et al., 2008; Fiske, 2018; Fiske et al., 2007; Wojciszke, 1994, 2005), a growing body of research suggests that they should be considered as independent dimensions (e.g., Brambilla et al., 2011, 2021; Brambilla & Leach, 2014; Goodwin, 2015; Goodwin et al., 2014; Luttrell et al., 2022; Wojciszke, 2005). Specifically, researchers have proposed that the warmth dimension should be divided into two sub-dimensions—sociability and morality—resulting in a three-dimensional model of person perception (Brambilla et al.,2011, 2021; Brambilla & Leach, 2014; Goodwin, 2015; Goodwin et al., 2014; Luttrell et al., 2022). Sociability here refers to character traits that facilitate forming connections and creating affectionate relationships, such as friendliness, likability, and extroversion, whereas morality encompasses characteristics that signal integrity and virtue, for example, honesty, sincerity, and trustworthiness (for reviews, see Brambilla & Leach, 2014; Goodwin, 2015).

Although there is experimental evidence that people evaluate traits corresponding to these two sub-dimensions of warmth differently, there are also traits that cannot easily be classified into either sub-dimension, as they are relevant to both sociability and morality. Examples include charitableness, generosity, and kindness (Goodwin et al., 2014). For the remainder of this paper, we focus on the cues to reputation that are shared between sociability and morality. Thus, we distinguish between cues that reveal something about a person’s competence to perform a particular task and cues that indicate their willingness to share and help others, which we refer to as “helpfulness.” These aspects of one’s reputation will, we argue, play a primary role in many everyday contexts when we must decide whether to cooperate with someone on the basis of their reputation.

### Reputation and Cooperation

In the context of cooperation, existing research on reputation predominantly focused on either the role of warmth traits (or sociability, morality, willingness to confer benefits) or competence (or ability to confer benefits) (Barclay, 2013; Fiske et al., 2007; Wojciszke, 2005). We review these two lines of research in turn, below.

#### Warmth/Morality

Overall, there appears to be a positive relationship between the character traits associated with warmth and cooperative behavior. For instance, several studies have shown that costly prosocial behaviors that require time and resources, such as volunteering or making charitable contributions, tend to attract interaction partners and result in long-term cooperation (e.g., André, 2010; Barclay, 2016; E. A. Smith & Bird, 2005). In addition, several field studies with hunter-gatherer communities, including the Hadza of Tanzania, Ache of Paraguay, and Quechua of Peru, have found that people tend to be more cooperative toward others who have a good reputation on warmth-related character traits, such as helpfulness and generosity (Barclay, 2013; Gurven et al., 2000, 2001; Lyle & Smith, 2014; Macfarlan et al., 2013; E. A. Smith, 2004; K. M. Smith & Apicella, 2020; K. M. Smith et al., 2022; Tognetti et al., 2014). Similar effects have been found even in non-human species: In an experimental study involving Japanese Quail fish (Ophir et al., 2005), females avoided aggressive fish and generally chose less aggressive male partners. When females were presented with equally kind (non-aggressive) males, they seemed indifferent in their partner choice (Ophir et al., 2005).

In incentivized studies using economic games, a partner’s past record of generosity and helpfulness has been demonstrated to affect a player’s behavior in joint tasks (Barclay, 2006; Barclay & Willer, 2007; Jordan et al., 2016; Van Vugt et al., 2007). For example, Wedekind and Braithwaite (2002) asked their participants to complete a Prisoner’s Dilemma on three consecutive days. On the first day, participants familiarized themselves with the experimental setup. On the second day, participants took part in a Prisoner’s Dilemma with several other participants (2–15, randomly determined). On Day 3, pairs of participants completed the Prisoner’s Dilemma again, but this time with knowledge about other players’ behavior on the preceding day. The authors found that more helpful partners were rewarded with higher levels of cooperation from other players, thus accumulating more money in the long term (Wedekind & Braithwaite, 2002). In a Public Goods Game, Milinski et al. (2002) showed that participants were more likely to cooperate with people who made larger contributions that benefited everyone else in the group (i.e., people who contributed to the public good). In a similar vein, Albert et al. (2007) explored whether players’ cooperative behaviors in the Prisoner’s Dilemma and investment game were influenced by the amount of their partners’ past charitable donations. The authors found that generous players were more likely to cooperate, but particularly with those who were also generous, thus showing that people are “nicer to nicer people” (see also Jordan et al. (2016)).

#### Competence

Paralleling the studies of warmth/morality, experimental and field studies have found a positive relationship between a partner’s competence and others’ willingness to cooperate with them. This positive relationship was, for instance, observed in field studies of hunter-gatherer communities, such as the Hadza of Tanzania, the Ache of Paraguay, and the Chabu in Ethiopia (e.g., Apicella, 2014; Apicella et al., 2012; Garfield et al., 2019; Gurven et al., 2000; E. A. Smith, 2004; K. M. Smith & Apicella, 2020; K. M. Smith et al., 2022; Von Rueden et al., 2014). These studies demonstrated that signaling competence through qualities, such as physical strength, hunting abilities, or farming skills led campmates to view individuals as more suitable partners. Similar results were also observed in controlled animal studies with chimpanzees, birds, and even fish (e.g., Doutrelant & McGregor, 2000; McGregor & Peake, 2000; Melis et al., 2006; Oliveira et al., 1998; K. Otter et al., 1999; K. A. Otter et al., 2001; Vail et al., 2014).

Incentivized experimental studies emerging from the field of behavioral economics have mostly centered around how a player’s own competence predicts whether or not they will cooperate with another individual (e.g., Alaoui & Penta, 2016; Burks et al., 2009; Gill & Prowse, 2016). Nonetheless, research that has focused on how a player’s cooperative behavior is determined by their knowledge about the potential partner’s competence has typically also found a positive relation between the judged competence of a partner and their willingness to cooperate with that partner (e.g., Heck & Krueger, 2017; Krueger, 2019; Krueger & Acevedo, 2007; Krueger et al., 2020; Lambrecht et al., 2024; Proto et al., 2019). For example, in a two-stage experiment, Proto et al. (2019) measured players’ competence in Stage 1 using the Raven’s Advanced Progressive Matrices test. In Stage 2, players participated in several different economic games, including Prisoner’s Dilemma, battle of the sexes, and stag hunt. In addition to their main finding that more competent players were more likely to behave cooperatively, the authors found that players preferred to cooperate with more competent partners (Proto et al., 2019). The same researchers investigated how a player’s cooperativeness differed with individuals who rank either high or low on intelligence (Lambrecht et al., 2024). A large disparity in intelligence between two players impeded cooperation in the early rounds of a repeated Prisoner’s Dilemma. These results were driven by those who were rated as more intelligent, who were less cooperative when interacting with a less intelligent partner, particularly in the condition when the information about all players’ intelligence was publicly disclosed (Lambrecht et al., 2024). Judgments of other players’ competence may also depend on both strategy selection and the outcome of social interactions. In their review of the literature, Krueger et al. (2020) showed that in the context of Prisoner’s Dilemma (where the dominant strategy is to defect), participants rated cooperators as less competent, but only if their partner also defected (Krueger & Acevedo, 2007). When the dominant strategy was no longer to defect (as in the Volunteer’s Dilemma), participants rated cooperators as more competent in general (Krueger, 2019) but rated those who both defected and had their partners defect as less competent (Heck & Krueger, 2017; Krueger et al., 2020). Thus, competence ratings depend not only on the chosen strategy but also on the final payoff. This finding differs from those in ratings of morality (broadly related to warmth, trustworthiness, and honesty, see Krueger [2019]), which were higher whenever a player’s decision benefited another person. These and other findings align with research on person perception, where the general positive–negative evaluation on the warmth dimension has priority over the judgment of how competent a person is (Fiske et al., 2007).

#### Warmth/Morality versus Competence

Taken together, experimental evidence suggests that people (and non-human animals) prefer to interact and cooperate with others whose reputation signals warmth and competence (Albert et al., 2007; Barclay, 2006, 2013; Barclay & Willer, 2007; Doutrelant & McGregor, 2000; Gurven et al., 2000, 2001; Heck & Krueger, 2017; Jordan et al., 2016; Krueger, 2019; Krueger & Acevedo, 2007; Krueger et al., 2020; Lambrecht et al., 2024; Lyle & Smith, 2014; Macfarlan et al., 2013; McGregor & Peake, 2000; Melis et al., 2006; Oliveira et al., 1998; K. A. Otter et al., 2001; Proto et al., 2019; E. A. Smith, 2004; K. M. Smith & Apicella, 2020; K. M. Smith et al., 2022; Tognetti et al., 2014; Vail et al., 2014; Van Vugt et al., 2007). However, the research outlined above has examined the effects of these traits on cooperative behavior in isolation. An outstanding question is how people trade off these two dimensions. Which traits do people value as more informative when judging and deciding how to interact with someone? In many everyday situations, one reputational cue may be more critical to successfully achieving a specific goal or task than the other. Some tasks may benefit from a highly competent partner, while in others, warmth is more beneficial.

Some insights into the interplay between different reputational cues come from studies concerned with cue prioritization in general evaluative judgments of others (e.g., Dores Cruz, Thielmann, et al., 2021; Dores Cruz, van der Lee, & Beersma, 2021; Henrich et al., 2010; Henrich & Gil-White, 2001). For example, a large body of evidence supports the so-called “primacy of warmth” or “primacy of morality” in impression formation. Accordingly, impressions tend to be more strongly influenced by variations in a person’s traits related to warmth (in a two-dimensional model) and morality (in a three-dimensional model) than by traits related to competence (for reviews, see Abele et al., 2021; Brambilla et al., 2021; Fiske et al., 2007; Koch et al., 2021; Wojciszke, 2005). Furthermore, people tend to view negative moral traits as more indicative of a person’s character than positive moral traits (Abele et al., 2021). Eisenbruch and Krasnow (2022) argue that people prioritize traits associated with warmth or morality because the variation in individuals’ traits on this dimension is greater than on the competence dimension, making it a more informative predictor of future behavior.

A number of studies have also examined how reputations for character traits in one domain influence judgments of character in the other domain (e.g., Abeler et al., 2019; Chen & Guo, 2021; Gräf & Unkelbach, 2016; Stellar & Willer, 2018). For instance, Chen and Guo (2021) found that high competence positively influences perceptions of a person’s traits on the warmth dimension. In this study, initial judgments of a person’s warmth were influenced by their competence: individuals originally perceived as cold were rated as warmer if they were also highly competent (Abeler et al., 2019; Chen & Guo, 2021).

Conversely, when individuals were initially perceived as warm, low competence led to a reduction in warmth judgments (Chen & Guo, 2021). Similarly, Stellar and Willer (2018) found that people perceived moral characteristics as more reliable predictors of behavior than traits associated with competence. However, some studies have found that character traits from different dimensions have an equal effect on people’s judgments and behavior (Dores Cruz, Thielmann, et al., 2021; Dores Cruz, van der Lee, & Beersma, 2021). In a more direct test of how people prioritize from multiple cues about one’s reputation, Hackel et al. (2022) compared the effect of trait-based learning and reward-based learning in impression formation. Across four experiments, participants were able to observe the performance of other participants on various tasks to learn about their mathematics and verbal skills. When presented with partner choice in a new task, participants were found to make generalizations about one aspect of competence (e.g., math) even in a new and unrelated context.

### Present Work

Building on the previous work about the multidimensional structure of reputation, here we investigate how people integrate information about different reputational cues. Reputation in our experiment is defined as the information we provide to each participant about the partner’s past behavior in a previous game. This behavior can be informative about either the warmth or the competence of a given player. We explore whether people pay selective attention to the most relevant reputational cue when deciding whether to send money to another person. To make one of the cues more relevant to the task at hand, we conducted three experiments in which our participants played a series of one-shot investment games with multiple different partners (the receivers). In Experiments 1 and 2, the amount sent was tripled before receivers decided how much of the money they were willing to return to the sender. In this context, as far as senders are concerned, the receiver’s past helpfulness (here, the cue to warmth) was a more relevant reputational cue than the receiver’s competence. In Experiment 3, the amount sent by a sender determined how many puzzles would be unlocked for the receiver to solve. Solving each puzzle earned a bonus, and the total earnings were equally split between the two players. Unsolved puzzles resulted in lost investment, and so the sender had to decide whether they felt confident that the receiver could solve puzzles and increase total earnings for both players. Thus, in this context, it was the receiver’s performance (here, cue to competence) reputation that was more relevant. In all three experiments, we analyzed senders’ willingness to send money as a function of receivers’ reputation on both the task-relevant and the task-irrelevant reputational cues. We hypothesized that people would be proficient in assessing their partner’s reputation. Following our results from Experiment 1, we expected to find positive main effects for the reputational cues and an interaction effect.^1^

Our work builds on the methods used by Hackel and Zaki (2018) and Hackel et al. (2022). Our approach differs from theirs in a number of ways. First, we examined the role of people’s varying character traits on two dimensions, namely warmth and competence. Second, we evaluated people’s decisions in incentivized economic games. That is, in our experiment, individuals’ judgments and interpretations of individual traits in new contexts had monetary consequences. Further, while we also use an adapted version of the game introduced by Berg et al. (1995), we investigated how the task context influences the behavior of a *sender*. We created very specific environments in which we control the task: each game is set up in such a way that one of the partner’s reputational cues is clearly prioritized, as it more strongly determines the successful outcome of the task than the other. Unlike previous work that focused on overall evaluation based on multiple cues (see studies on the primacy of warmth/morality, for example), our research, therefore, tests the conditions in which participants must identify a task-relevant cue, ignoring irrelevant information about a partner’s reputation.

## Experiment 1

### Methods

#### Design

**Figure 1.**
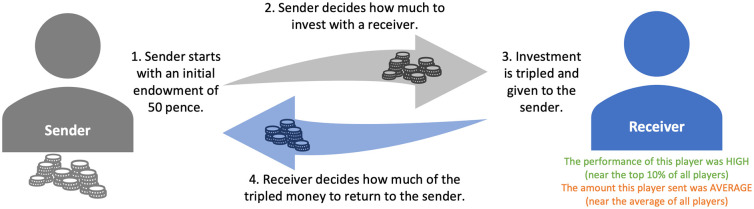
Setup of Experiment 1. *Note.* Senders start with a 50 pence endowment and decide how much to invest by sending some money to the receiver. The invested amount is tripled before being given to the receiver. The receiver then chooses how much of the tripled investment amount to return to the sender.

In Experiment 1, the effect of reputation was tested using a modified investment game. In each round of the game, participants decided how much money to send to a receiver, with the hope that the receiver would return their money (perhaps even with some profit) at a later stage. Since our focus was on senders’ decisions, we chose not to pair senders and receivers in real-time. Instead, on each round, a sender decided whether they would be willing to send money to a receiver who was described to them in terms of their reputation for competence and helpfulness. The information was presented in terms of categories, where each receiver could be described as low, medium, or high on each cue. Each combination of cues could appear twice, resulting in 18 trials in total (9 types of cue combinations × 2). Receivers with different reputations were shown to each sender in random order, and the amount of money that senders were willing to send on each round was our main dependent variable. Figure 1 shows an overview of the design for the investment game.

In addition to collecting data from senders, we allocated a small group of our participants to the role of receivers (see “Participants” section). These receivers made only a single decision, indicating how much (proportionally) of the total pot of money they would be willing to send back to a sender. We used these data for the purpose of incentivizing senders’ decisions. Specifically, we matched each sender to one receiver at the end of the study to calculate their final payoffs based on a randomly selected round of the investment game.

Experiment 1 began with a pretest section. All participants, including those who were eventually allocated to the group of senders or receivers, completed this pretest. During this pretest, all participants completed two tasks that were designed to measure their own helpfulness and their competence (see below for details). This stage had two purposes. First, completion of the two tasks allowed those who would eventually become senders in the investment game to learn how the reputation of receivers is determined. Second, we used responses from the group of participants who were eventually allocated to be receivers to determine whether they were high, medium, or low on each reputational cue, which was necessary for matching senders to receivers for the purpose of incentivization.

#### Participants

For Experiment 1, 140 participants were recruited through the online recruitment platform Prolific. We recruited participants who had an approval rating above 90% and were fluent in English. All participants were given a fixed participation fee of more than £5 per hour for an average of 10–13 minutes of their time (depending on their player type). All participants could earn an additional bonus based on their performance in the matching task (see below) and their choice in one randomly selected round of the investment game.

We collected responses for both types of players, those taking on the role of the sender (*N =* 100) and the receiver (*N =* 40). One response from a participant facing technical difficulties in the receiver group was excluded, resulting in a final sample of 39 participants (27 women, 8 men; *M*_age_ = 26.35, *SD* = 7.60). To answer our research question, our analysis concentrated on responses from participants (*N =* 100) in the sender role (72 women, 25 men; *M*_age_ = 27.95, *SD* = 9.76).

#### Pretest

In the pretest, we used a matching game to determine helpfulness and competence reputation of the receivers.

##### Helpfulness Reputation

Before completing the matching game (but after learning the rules of the game), participants were informed that they were randomly paired with another player and that this player could not earn any bonus from completing the matching game. Participants were given an opportunity to decide whether they would be willing to share some of their earnings from the matching game with that player. This could be anywhere between none (0%) and all (100%) of the money they would earn.

##### Competence Reputation

In the matching game, participants were presented with multiple 3 × 4 matrices containing 12 numbers rounded to one decimal place. Each matrix contained one pair of numbers that sum up to 10 (e.g., 1.8 and 8.2). Participants were asked to find as many number pairs as possible within a 3-minute time limit. If they could not find the pair in a matrix, they could skip it without being penalized. The number of matrices presented exceeded the number that anyone would reasonably complete (to prevent any anchoring effects about the number of matrices participants believed they were expected to solve within the given time). Participants received a bonus for each correctly matched number pair minus the percentage they chose to share with another person. Competence was measured by the relative number of correctly solved matrices in the time provided.

#### Investment Game

After the pretest was completed, participants proceeded to the main part of the experiment, which consisted of multiple investment games (Berg et al., 1995). This was the main part of the experiment, during which some participants were allocated to the group of senders, and some were receivers. Those who were allocated to the group of senders played 18 one-shot rounds of the investment game, each with 9 different types of receivers. At the start of a round, the sender was given an initial endowment of 50 pence. The sender then had to decide how much of their money they wanted to send to the receiver, which was then tripled. For example, if the sender sent 10 pence, the sender would have 40 pence left, and the receiver would have 30 pence at their disposal. Senders were aware that any receiver could return some fraction of the enlarged pot to them. Since the investment game was not played out in real-time, senders did not receive immediate feedback about the amount of money a receiver would send them back and were instead told that their payoffs were going to be calculated at a later stage after the experiment was concluded.

A critical element of the investment game was that, on each round, senders were presented with two reputational cues about the receiver. On each round, they were told that their receiver was high/medium/low in terms of their helpfulness and competence, as determined by their behavior in the pretest (i.e., in the matching game). The helpfulness acted as a cue to warmth and was task-relevant since the sender could only rely on the receivers’ willingness to share money with them. Receivers’ performance on the matching game (cue to competence), however, was not relevant.

As a reminder, note that those who were allocated to the group of receivers (40 participants) were only asked what proportion of money they would be willing to send back to a sender.

#### Incentive Structure

Participants’ bonuses depended on several factors. First, all participants could forfeit some of their money by choosing to share their winnings from the matching game during the pretest. Depending on their performance in the game (i.e., their competence) and their willingness to share with another person (i.e., their helpfulness), their earnings from this part could range from 0 to £1.50.

In addition, senders’ decisions were incentivized by pairing them with a random receiver at the end of the game.^2^ One of these rounds was eventually chosen to calculate bonus payments. For this round, we chose from the group of 40 receivers a person whose reputation matched the reputation of the person that a sender was paired with on that trial. We achieved this by matching the low/medium/high to the 10th, 50th, and 90th percentile of helpfulness and competence scores in the full sample from the pretest. We then used the chosen receiver’s decision about what percentage of the money to send back to the sender to calculate the final earnings from the investment game for a given sender.

### Results

We tested the effect of the receivers’ reputations on senders’ levels of investment using a mixed-effects model. This model allowed us to account for the within-participant structure of the data, whereby participants saw all combinations of reputational cues and were given the opportunity to invest money with each type of receiver twice (Singmann & Kellen, 2019). Our model included fixed effects of the receiver’s reputation; a three-level factor for competence (low, medium, high), a three-level factor for helpfulness (low, medium, high), and the interaction between the two. We simplified the maximal model until our model converged (Bates et al., 2015), which resulted in a final model that included random slopes and random intercepts for the helpfulness and competence reputations of each receiver.^3^ The regression equation for our final model is: *Sender’s Investment Amount_ij_ = β_0_ + β_1_ Receiver’s Helpfulness_ij_ + β_2_ Receiver’s Competence_ij_ + β_3_ (Receiver’s Helpfulness_ij_ Receiver’s Competence_ij_) + u_0j_ + u_1j_ Receiver’s Helpfulness_ij_ + u_2j_ Receiver’s Competence_ij_ + ε_ij_*.^4^ We report our *p*-values for all fixed-effect models obtained from the afex (Singmann & Kellen, 2019) package in R and partial η^2^_*p*_ obtained using the effect size package.

**Figure 2.**
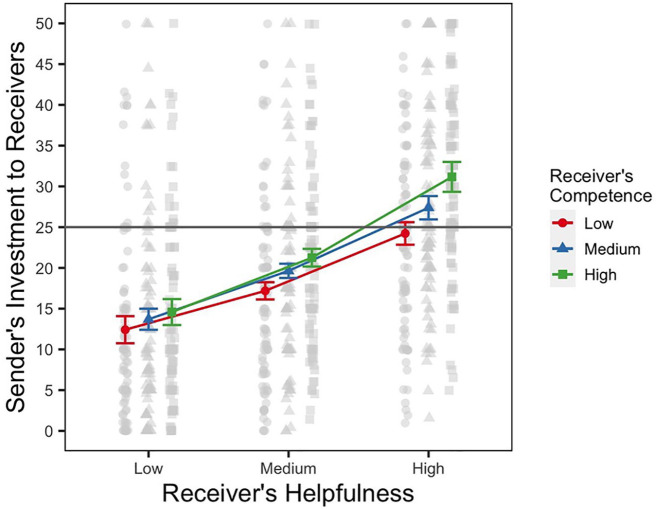
Results of Experiment 1. *Note.* Figure shows the marginal means for the amounts sent by senders to receivers from the random effects model. The figure includes within-subject (Cousineau & O’Brien, 2014) error bars of the sender’s level of investment to each receiver. The raw data of the sender’s level of investment are shown in gray. The gray horizontal line signifies half of the sender’s endowment. The *x*-axis shows three reputation levels representing the receiver’s helpfulness (low, medium, and high), and the outcome-relevant cue in this experiment.

The results of our mixed-effects model show a significant main effect of helpfulness *F*(2, 100) = 77.24, η^2^_*p*_ = .61, *p < .*001 and of competence: *F*(2, 102) = 25.61, η^2^_*p*_ = .33, *p* < .001. The marginal means for each cell of our design, along with the raw data, are shown in Figure 2. Overall, we found that senders invested more money when their partner had a reputation for being more helpful. We find that on average, senders sent 13.6 pence (*SE* = 1.15, 95% CI = [11.31, 15.83]), 19.4 pence (*SE* = 1.03, 95% CI = [17.33, 21.39]), and 27.6 pence (*SE* = 1.26, 95% CI = [25.31, 29.87]) to receivers who were described as scoring low, medium, and high on helpfulness, respectively. Competence played a lesser role, judging from the effect sizes and the pattern of means. Averaging over helpfulness ratings, senders invested 17.9 pence (*SE* = 1.04, 95% CI = [15.88, 20.00]), 20.24 pence (*SE* = 1.02, 95% CI = [18.21, 22.27]), and 22.33 pence (*SE* = 1.05, 95% CI = [20.25, 24.41]) with receivers who were described as having low, medium, and high competence, respectively. Crucially, we also found a significant interaction, with *F*(4, 1,317) = 6.86, η^2^_*p*_ = .02, *p* < .001, indicating that the effect of helpfulness varied as a function of receivers’ competence. As can be seen in Figure 2, senders sent more money when the receivers had a reputation of being highly helpful *and* highly competent. Although this effect is relatively small, pairwise comparisons of money sent, which are summarized in Table 1, show that the largest differences between competence levels can be observed when the receiver had a reputation of being most helpful. To test this effect more formally, we computed additional contrasts to compare the differences between money sent to the most competent and least competent receiver. Comparing the difference in money invested between low and high competence reputation when the receiver is most helpful with the difference when the receiver has a reputation of low helpfulness (i.e., Rows 2 and 8 in Table 1) yields a significant difference, with an estimate of −4.77 (1,315), *SE* = .93, *t* = −5.12, *p* <.001. The difference is also significant if we compare the high and medium helpfulness, that is, Rows 8 and 5 in Table 1: −2.88 (1,315), *SE* = .93, *t* = −3.08, *p* =.002. Together, these results indicate that as the receivers’ helpfulness reputation increases, their reputation on the competence cue begins to play a bigger role in senders’ investment decisions.

**Table 1.** Experiment 1 Results for Follow-Up Pairwise Comparison Tests.

Receiver’s Helpfulness Reputation	Receiver’s Competence Reputation	Estimate	*SE*	*df*	*t*	*p*
Low	Low–Medium	−1.29	.72	1161	−1.80	.219
	Low–High	−2.17	.82	300	−2.64	.026
	Medium–High	−.89	.76	424	−1.16	.738
Medium	Low–Medium	−2.46	.72	1161	−3.43	.002
	Low–High	−4.07	.82	300	−4.94	<.001
	Medium–High	−1.61	.76	424	−2.11	.106
High	Low–Medium	−3.16	.72	1161	−4.41	<.001
	Low–High	−6.94	.82	300	−8.43	<.001
	Medium–High	−3.79	.76	424	−4.97	<.001

Dependent variable: Senders’ level of investment.

Degrees-of-freedom method: Kenward–Roger.

*p-*value adjustment: Bonferroni method for three tests.

### Discussion

The results of Experiment 1 show that people are willing to invest more money when they know that the person who will be deciding how to split the earnings has a reputation for being helpful. Specifically, senders were more inclined to invest when they knew that, in the pretest, the receiver had shared some of their earnings from the matching game with a stranger. Participants in our study generally discounted information about the receivers’ competence. However, the interaction between the two cues reveals that both cues influence senders’ decisions, particularly when they are both high. In other words, people were more willing to invest when the receiver was helpful and competent than when a receiver was helpful but not competent. Our follow-up analysis revealed that participants asymmetrically evaluate competence depending on the receiver’s helpfulness. The competence of a receiver did not matter very much at lower levels of helpfulness, in contrast, showing that even being a very competent individual does not compensate (or does so to a much lesser extent) for a low helpfulness reputation. Compared with low competence, high competence did increase sender’s investment amounts for receivers with a high helpfulness reputation.

In Experiment 2, we set out to replicate our results of Experiment 1 with a different method of determining the receivers’ reputations.

## Experiment 2

In Experiment 2, we constructed reputation scores for our receivers based on their self-reported characteristics. More specifically, for the competence reputation, we asked our participants about their performance on the Scholastic Assessment Test (SAT), the standardized exam required to enter U.S. universities. For the helpfulness reputation, we asked participants about their past charitable engagement.

The design, hypotheses, and analysis plan for Experiment 2 were preregistered on the OSF repository (https://osf.io/h9nyu).

### Methods

#### Design

The structure of the investment game was identical to that of Experiment 1. The only difference between the two experiments was the design of the pretest, during which the reputation of our participants was determined.

During the pretest, participants’ helpfulness reputations were estimated by assessing their relative charitable contributions, asking them to respond to the question “How charitable are you in general? (For example, how much money do you give, how much time do you spend volunteering)” on a scale from 0 (*very little*) to 100 (*very much*). For the competence reputation, participants were asked three questions: to the questions, “Have you heard of the SAT?” and “Have you taken the SAT?” they were asked to provide a binary response (yes/no); finally, we asked participants “What total SAT score (out of 1600) did you get?”^5^

#### Participants

Building on the findings from Experiment 1, we performed a power analysis using Cohen’s *f* (Cohen, 1988) to determine how many participants in the role of a sender are needed to detect an interaction effect between the two reputation cues, as in Experiment 1. The analysis indicated a required sample size of 252 participants to achieve .9 power to detect a medium effect size of .28 at the standard .05 level of alpha.

In total, 302 participants were recruited through the online recruitment platform Prolific, comprising 252 participants in the role of sender. In addition to the previously outlined recruitment criteria above—having an approval rating above 90%, being fluent in English, and having no prior involvement in our previous study—we further limited our participant pool to U.S. residents (because we relied on the SAT score for competence reputation). In addition, we collected a sample of 50 participants (36 women, 14 men; *M*_age_ = 31.24, *SD* = 12.53) to take on the role of the receiver (once again, for the purpose of incentivization). All participants received a fixed show-up fee of £0.50 if they took on the role of the sender and £1 if they were receivers, as the time to complete the experiment for receivers was twice as long as for senders. All earned bonus money based on their choices in one randomly selected round of the investment game.

From our original sample of 252 participants who assumed the role of the sender in the investment game, two participants reported never having heard of the SATs and responded to our open-ended question that their data should be discarded. Consistent with our preregistration, these participants were excluded from our analysis and replaced with new participants. This process was carried out prior to any analysis or further processing of the data. Our final sample of senders aligned with our target sample size of 252 (178 women, 72 men; *M*_age_ = 35.66, *SD* = 13.07).

### Results

As in Experiment 1, the maximal converging mixed-effects model included random effects (by-participant slopes and by-participant intercepts) for the receiver’s reputation for helpfulness. Results of non-converging models, and additional analyses including senders’ own helpfulness and competence from the pretest, are reported in the Supplement (Appendix B), but here we note that they do not alter any findings reported below.

**Figure 3.**
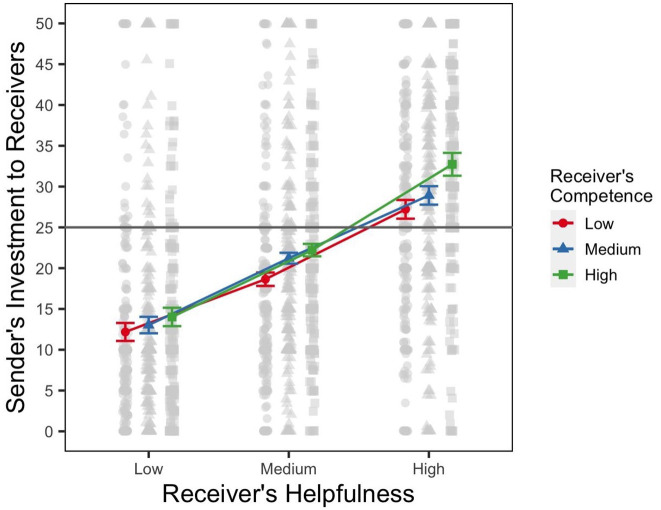
Results of Experiment 2. *Note.* Figure shows the marginal means for the amounts sent by senders to receivers from the random effects model. The figure includes within-subject (Cousineau & O’Brien, 2014) error bars of the sender’s level of investment to each receiver. The raw data of the sender’s level of investment are shown in gray. The gray horizontal line signifies half of the sender’s endowment. The *x*-axis shows three reputation levels representing the receiver’s helpfulness (low, medium, high), the outcome-relevant cue in this experiment.

Consistent with Experiment 1, the results of our mixed-effects model show a significant main effect of helpfulness, *F*(2, 254) = 214.62, η^2^_*p*_ = .63, *p* < .001, and of competence, *F*(2, 3,779) = 143.19, η^2^_*p*_ = .07, *p* < .001. The marginal means, along with raw data, are summarized in Figure 3 and reveal a familiar pattern. As in Experiment 1, we found that senders invested more money when the receiver had a reputation of being more helpful. We find that on average, senders sent 13.1 pence (*SE* = .84, 95% CI = [11.43, 14.72]), 20.7 pence (*SE* = .77, 95% CI = [19.18, 22.20]), and 29.62 pence (*SE* = .86, 95% CI = [27.94, 31.30]) to receivers who were described as scoring low, medium, and high on helpfulness, respectively. As in Experiment 1, competence played a lesser role, as judged from the effect sizes and the pattern of means. Averaging over helpfulness ratings, senders invested 19.3 pence (*SE* = .75, 95% CI = [17.87, 20.82]), 21.05 pence (*SE* = .75, 95% CI = [19.57, 22.53]), and 22.99 pence (*SE* = .75, 95% CI = [21.51, 24.46]) with receivers who were described as having low, medium, and high competence, respectively. In addition, we found a significant interaction, with *F*(4, 3,779) = 16.27, η^2^_*p*_ = .02, *p* < .001, which indicates that the effect of receiver’s helpfulness on sender’s investment behavior varied as a function of receivers’ competence. Judging from Figure 3, we can once again see that senders sent more money when the receivers had a reputation of being highly helpful *and* highly competent. Pairwise comparisons of money sent, which are summarized in Table 2, show a pattern consistent with Experiment 1. Comparing the difference in money invested between low and high competence reputation when the receiver is most helpful with the difference when the receiver has a reputation of low helpfulness (i.e., Rows 2 and 8 in Table 2) shows a significant difference, with an estimate of −3.68 (3,778), *SE* = .53, *t* = −6.98, *p* <.001. The difference is also significant if we compare the high and medium helpfulness, that is, Rows 8 and 5 in Table 2: −1.95 (3,778), *SE* = .53, *t* = −3.70, *p* <.001. Together, these results replicate the findings from Experiment 1: as the receivers’ helpfulness reputation increases, senders’ investment decisions are more strongly influenced by their reputation on the cue for their competence.

**Table 2.** Experiment 2 Results for Follow-Up Pairwise Comparison Tests.

Receiver’s Helpfulness Reputation	Receiver’s Competence Reputation	Estimate	*SE*	*df*	*t*	*p*
Low	Low–Medium	−.85	.37	3778	−2.27	.071
	Low–High	−1.84	.37	3778	−4.93	<.001
	Medium–High	−.99	.37	3778	−2.66	.023
Medium	Low–Medium	−2.57	.37	3778	−6.90	<.001
	Low–High	−3.57	.37	3778	−9.57	<.001
	Medium–High	−1.00	.37	3778	−2.67	.023
High	Low–Medium	−1.70	.37	3778	−4.56	<.001
	Low–High	−5.52	.37	3778	−14.80	<.001
	Medium–High	−3.82	.37	3778	−10.23	<.001

Dependent variable: Senders’ level of investment.

Degrees-of-freedom method: Kenward–Roger.

*p-*value adjustment: Bonferroni method for three tests.

### Discussion

Results of Experiment 2 closely replicate our findings from Experiment 1. Although our participants clearly pay more attention to the task-relevant cue of helpfulness, they are nonetheless influenced by the cues to receivers’ competence reputation. Our findings show that the results of Experiment 1 were not due to the nature of the pretest; even reputation based on self-reported charitable donations and SAT scores leads to the same pattern of senders’ decisions.

## Experiment 3

In Experiment 3, we tested whether the pattern observed in Experiments 1 and 2 holds true when the task-relevant reputational cue is competence rather than helpfulness.

The experimental design, hypotheses, and analysis plan for Experiment 3 were preregistered on OSF (https://osf.io/srvgx).

### Methods

#### Design

The key new feature of Experiment 3 was the use of a different type of investment game. The design of the pretest, necessary to establish participants’ competence and helpfulness reputation, was identical to that of Experiment 1.

During the new investment game, senders were given an initial endowment of £1 and were offered the chance to send some of it to the receiver. The chosen amount determined the number of puzzles unlocked for the receiver to solve. The amount of money that senders could send was in increments of 10 pence. In other words, every 10 pence the sender sent unlocked one additional puzzle that the receiver could try to solve. Senders were allowed to spend any portion of their endowment to unlock the puzzles, ranging from spending everything to unlock all 10 puzzles to keeping all of their money and not paying to unlock any puzzles at all.

Senders were informed that receivers would have 2 minutes to solve as many puzzles as they could. For each correctly solved puzzle, the receiver earned a bonus of 20 pence. The amount earned by the receiver would be equally split between the players automatically. In addition, the sender would be reimbursed for their 10-pence investment for that puzzle. For example, if a sender decided to send 30 pence to the receiver, they would definitely get to keep the remaining 70 pence, and their investment unlocked three puzzles for the receiver to attempt. If the receiver correctly solved two puzzles, they earned 40 pence, which would be evenly split between the two players. In the end, the receiver would get a bonus payment of 20 pence from this game. The sender would earn a bonus of £1.10 comprising the initial 70 pence they did not send, the 20 pence bonus from correctly solved puzzles, and the 20 pence reimbursement of what unlocking the correctly solved questions cost them.

Incentives were calculated in the same way as in Experiment 1, whereby one receiver was paired with each sender based on a randomly selected round of the game. Based on the receiver’s true performance in the matching game (from the pretest), the payout for both players was calculated.

#### Participants

Relying on the power analysis from Experiment 2, our aim was to collect data from 252 participants in the sender role. Consistent with the inclusion criteria applied in Experiment 1, Prolific participants were required to have an approval rating exceeding 98%, be fluent in English, and have no prior involvement in our previous studies. From the initial sample of 252 participants taking on the role of the sender, 17 participants were excluded as they indicated that they either did not understand the experiment or were distracted (as per our preregistration). These missing responses were replaced with responses from new participants, allowing us to reach our final target size, recruiting 254 participants (121 women, 130 men; *M*_age_ = 37.45, *SD* = 13.63). We also collected data from 50 participants taking on the role of receivers for subsequent pairing with senders and calculating their bonus payment. One receiver from the original sample was replaced to achieve the sample of 50 participants (33 women, 16 men; *M*_age_ = 34.35, *SD* = 12.27).

### Results

**Figure 4.**
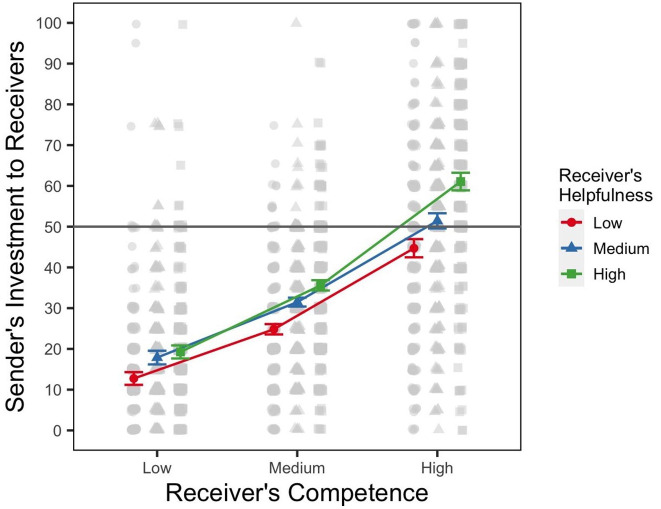
Results of Experiment 3. *Note.* Figure shows the marginal means for the amounts sent by senders to receivers from the random effects model. The figure includes within-subject (Cousineau & O’Brien, 2014) error bars of the sender’s level of investment to each receiver. The raw data of the sender’s level of investment are shown in gray. The gray horizontal line signifies half of the sender’s endowment. The *x*-axis shows three reputation levels representing the receiver’s competence (low, medium, and high), the outcome-relevant cue in this experiment.

As for previous experiments, here we report the results of the maximal converging model, which included random effects (by-participant slopes and by-participant intercepts) for each receiver’s helpfulness and competence reputations, with additional analyses reported in the Supplementary Materials (Appendix A). The results of our mixed-effects model show a significant main effect of competence, *F*(2, 256) = 335.32, η^2^_*p*_ = .72, *p* < .001; and helpfulness: *F*(2, 262) = 103.33, η^2^_*p*_ = .44, *p* < .001. The marginal means, summarized in Figure 4, show that the pattern observed in Experiment 3 mirrors that seen in Experiments 1 and 2. Once more, we find that senders pay more attention to the relevant cue (here competence, on the *x*-axis), investing more when their receiver is rated as highly competent. We find that on average, senders sent 16.6 pence (*SE* = 1.16, 95% CI = [14.34, 18.91]), 30.6 pence (*SE* = 1.01, 95% CI = [28.64, 32.62]), and 52.4 pence (*SE* = 1.20, 95% CI = [50.03, 54.76]) to receivers who were described as scoring low, medium, and high on competence, respectively. Helpfulness mattered less—averaging over competence ratings, senders invested 27.4 pence (*SE* = 1.02, 95% CI = [25.41, 29.44]), 33.6 pence (*SE* = .97, 95% CI = [31.69, 35.49]), and 38.6 pence (*SE* = 1.02, 95% CI = [36.62, 40.65]) with receivers who were described as having low, medium, and high helpfulness, respectively. Crucially, we also found a significant interaction effect between competence and helpfulness, *F*(4, 3,289) = 32.35, η^2^_*p*_ = .04, *p* < .001. Figure 4 illustrates that senders sent more money when the receivers had a reputation of being highly competent *and* highly helpful. Pairwise comparisons are summarized in Table 3 and show that the difference in amount sent between the most and least helpful receivers was larger at high level of competence (i.e., Row 8 in Table 3) than at the low level of competence (i.e., Row 2 in Table 3), with the estimated contrast of −9.84 (3336), *SE* = .94, *t* = −10.47, *p* <.001. The difference is also significant if we compare high and medium competence, that is, Rows 8 and 5 in Table 3: −5.59 (336), *SE* = .94, *t* = −5.95, *p* <.001. Together, these results indicate that as the receivers’ competence reputation increases, their reputation on the helpfulness cue begins to play a bigger role in senders’ investment decisions.

**Table 3.** Experiment 3 Results for Follow-Up Pairwise Comparison Tests.

Receiver’s Competence Reputation	Receiver’s Helpfulness Reputation	Estimate	*SE*	*df*	*t*	*p*
Low	Low–Medium	−5.14	.75	2229	−6.86	<.001
	Low–High	−6.52	.96	543	−6.82	<.001
	Medium–High	−1.38	.75	1351	−1.83	.201
Medium	Low–Medium	−6.65	.75	2229	−8.89	<.001
	Low–High	−10.77	.96	543	−11.27	<.001
	Medium–High	−4.11	.75	1351	−5.47	<.001
High	Low–Medium	−6.71	.75	2229	−8.96	<.001
	Low–High	−16.36	.96	543	−17.12	<.001
	Medium–High	−9.65	.75	1351	−12.83	<.001

Dependent variable: Senders’ level of investment.

Degrees-of-freedom method: Kenward–Roger.

*p*-value adjustment: Bonferroni method for three tests.

### Discussion

The results of Experiment 3 are consistent with the results of Experiments 1 and 2, showing that participants are sensitive to the task-relevant reputational cue. Participants in our study were willing to invest more money when they knew that their partner was competent, but even more so if they knew that their partner was competent and helpful. Helpfulness of a receiver mattered even though earnings from the modified investment game were split equally between the players. The influence of receivers’ helpfulness reputation on investment amounts was asymmetrical, with a larger effect when the receiver’s competence was high than when it was low.

## General Discussion

The capacity to work together has been instrumental in the evolution of human societies, enabling us to build complex social structures and achieve collective goals. Cooperation and trust are, therefore, hallmarks of human society. In the modern world, where we no longer live in small, close-knit communities and often must interact with strangers, reputation serves a critical function in bridging information gaps (Berg et al., 1995; Brandt et al., 2003; Brandt & Sigmund, 2004; Nowak, 2006; Nowak & Sigmund, 1998; Ohtsuki & Iwasa, 2004, 2006; Seinen & Schram, 2006; van Dijk & De Dreu, 2021). It is, therefore, reasonable to expect people to be adept at assessing reputation and making decisions based on such assessments. In the present paper, we hypothesized that people would demonstrate flexibility and proficiency in assessing specific, task-relevant dimensions of others’ reputations.

Our results were consistent with this broad hypothesis. In three experiments with monetary incentives, we analyzed the behavior of participants taking on the role of the sender in adapted versions of the investment game (Berg et al., 1995). Our design allowed us to determine whether the investment amounts varied in response to the reputation of the receivers. Our results consistently demonstrated that senders made investment decisions primarily based on the receiver’s most task-relevant reputation characteristic, paying less attention to the secondary reputation information in each experiment. However, we also found that the other cue still exerted a small influence over senders’ decisions, particularly when a receiver already scored highly on the task-relevant cue. This asymmetric spillover of reputation means that people are more likely to invest when their partner is both competent and helpful, irrespective of the receiver’s role in the investment game. Nevertheless, our findings consistently indicate that when a receiver ranks low on the outcome-relevant reputation cue, the influence of the other cue on the sender’s investment choices is negligible.

The interaction effect observed across three studies can be interpreted through the lens of research on the “primacy of warmth judgments” (Cacioppo et al., 1997; Eisenbruch & Krasnow, 2022; Peeters, 2002; Reeder et al., 2002; Stellar & Willer, 2018; Wojciszke et al., 1993, 1998). An important feature of warmth is that it acts as the primary basis of evaluation—a group or an individual needs to meet a minimum warmth threshold before competence can shape people’s reactions (Brambilla et al., 2011; Cacioppo et al., 1997; Goodwin et al., 2014; Peeters, 2002; Stellar & Willer, 2018). In other words, low warmth can reduce any positive impact of high competence, whereas high warmth creates a “halo effect,” causing further positive competence attributions (Gräf & Unkelbach, 2016; Judd et al., 2005; Rosenberg et al., 1968). The fact that competence mostly matters when warmth is high confirms that people first require some minimum threshold of warmth before factoring incompetence into their decisions, just as the models of person perception predict.

Our results also align with findings that judgments on one cue influence judgments on another cue. For example, warm individuals are perceived as more competent, and vice versa (Abeler et al., 2019; Chen & Guo, 2021). Such halo effects (Nisbett et al., 1977; Thorndike, 1920) may be ecologically grounded if distinct traits of people’s reputation are assumed to be correlated, providing a prior for evaluative judgments. Indeed, one can think of everyday examples when this happens, such as when tech leaders are given a platform to advise on moral issues (e.g., Elon Musk and Twitter takeover) or when charitable contributions of billionaires are overstated. Whether factually correct or not, it is surprising that a spillover can occur even if only one cue is task-relevant in an incentivized cooperative interaction with a stranger. Interestingly, our own data show that the two traits may not be strongly correlated. Although not preregistered, we calculated correlations between senders’ own levels of helpfulness and competence. We found no significant association for Experiment 1 but small and significant correlations in Experiments 2 and 3 (see Appendix B). This suggests that more competent individuals were actually less likely to help others, as indicated by the answers on our pretest.

Future work may explore situations where such an assumed correlation is or is not present. For example, it could be interesting to know whether people are able to learn from experience to ignore cues to warmth of a highly competent individual, when warmth has no impact on partners’ actual behavior. Since our studies did not provide immediate feedback, we cannot be sure whether the asymmetric spillover of reputation that we observed could be reduced when it carries either no or even negative value for an individual.

Although our use of economic games was motivated by the need to clearly separate task-relevant from task-irrelevant cues, the design of our studies is not without limitations. In many everyday contexts, cooperative decisions likely require consideration of multiple task-relevant cues. How individuals trade off different aspects of one’s reputation is an interesting research question, and our design cannot answer it. Nonetheless, the novelty of our result rests in the fact that even in context when one cue must be prioritized, there is evidence that decision-makers integrate information from the task-irrelevant cue into their decisions.

It is also worth noting that our asymmetric effect of two cues cannot be attributed to a floor effect, as even in the cases where receivers had a reputation of low competence and low helpfulness, our senders were willing to invest some of their money. Also, participants in our studies were generally cautious in investing, as the amounts sent (on aggregate) were at most only slightly higher than half of their endowments.

Our reputational cues were presented using verbal labels (low, medium, high), not with specific results describing the strangers’ behavior from the pretest. Senders were, therefore, free to interpret just how each reputation would translate into the receiver’s past behavior. Our results demonstrate that there is relatively little variance in our data. For example, consider the amounts sent to receivers with medium competence in Experiment 3. Here, our participants sent 24.8 pence on average (*SE* = 1.13) when receivers had a low reputation for helpfulness, 31.5 pence (*SE* = 1.08) when they had a medium reputation for helpfulness, and 35.6 pence (*SE* = 1.14) when the reputation for helpfulness was high.

**Figure 5.**
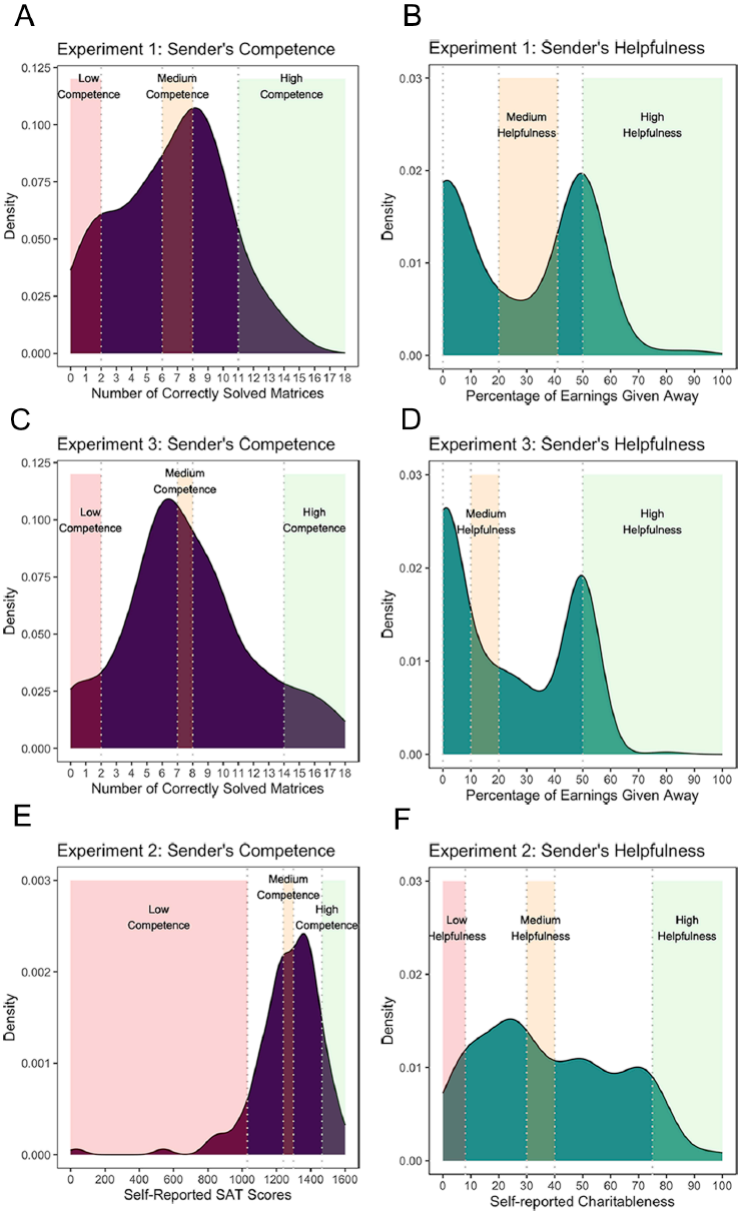
Distribution of Sender’s Character Traits *Note.* Distribution of senders’ level of competence (left columns: A, C, E) and helpfulness (right columns: B, D, F) for each experiment. The top two rows show the reputations for the experiments in which characteristics were elicited through tasks and choices in the pretest (Experiments 1 and 3), the bottom row represents self-reported characteristics (Experiment 2). Notably, panel E represents a reduced dataset, excluding participants who did not respond. Each reputation group (i.e., low, medium, and high) is identified through the dotted cut-off line and the shaded area. Panels B and D do not display a shaded region given that the cut-off for senders with a low helpfulness reputation was to share 0% of their earnings with a stranger.

Recall that our assignment to low, medium, and high categories was based on the distributions of participants’ decisions during the pretest. How helpful and how competent were our participants? Figure 5 summarizes the distributions obtained across the three experiments on both helpfulness and competence. The distributions within each category were comparable when these data were based on people’s actual decisions to share money and solve their performance in solving puzzles (as seen in Experiments 1 and 3, Figure 5A–D). However, when traits were self-reported (Experiment 2, Figure 5E and F), we find much more skewed distributions, with higher helpfulness and competence scores. It is also interesting that our senders were not particularly helpful (often opting to send 0 or only 50% future earnings) or competent (rarely solving 10 puzzles). It is plausible that our participants simply believed the receivers would behave similarly—sending back nothing or a maximum of half of the endowment. In addition, the limited number of participants who were able to solve 10 matrices might explain their cautious behavior in unlocking puzzles for the receiver.

Taken together, our results show that decision-makers carefully choose to cooperate more with helpful partners when they need someone who will be willing to share money with them, and a competent one when their earnings are tied to the partner’s ability to perform on a skill-based task. Yet, even task-irrelevant cues exert influence on people’s cooperative decisions, showing highest levels of cooperation toward highly competent *and* helpful individuals. The real-world consequences of such judgments can have serious consequences for individual welfare if misplaced. We hope that our work paves the ground for future research on the interplay between (a) features of the social context in which people must find a suitable cooperator and (b) multiple cues that signal strangers’ potential as a good cooperator.

## Supplemental Material

sj-docx-1-psp-10.1177_01461672241301116 – Supplemental material for Multiple Reputations: Selective Attention to Competence and Character Supplemental material, sj-docx-1-psp-10.1177_01461672241301116 for Multiple Reputations: Selective Attention to Competence and Character by Alexandra V. T. de La Trobe, Gordon D. A. Brown and Lukasz Walasek in Personality and Social Psychology Bulletin

## Notes

1. These hypotheses for Experiment 1 were based on previous findings in the literature. This, together with the results of Experiment 1, formed the bases for our Experiments 2 and 3, which we preregistered on OSF and provided more detailed hypotheses (for Experiment 2, see https://osf.io/h9nyu and for Experiment 3 https://osf.io/srvgx). Data and analysis code for all three experiments can be found at https://osf.io/279px/

2. Note that it was not necessary to pair senders with a receiver on every single round. For all practical purposes, only one receiver was needed, given that the senders’ earnings were based on a single randomly chosen round of the investment game.

3. Due to a singular fit in the maximal model, we first dropped the correlations between random effects from the model. When this did not work, we next dropped the random effects for the interaction term, which led to a successfully converging model. When comparing all three models, we found that the pattern of significant results was the same, with only slight variations in the obtained coefficients. Results from the other two models are available in the supplementary material (Appendix A). In addition, we tested an extended model in which we also included two covariates: the sender’s own competence and helpfulness established during the pre test. These results are not reported in the main manuscript but can be found in the supplementary material (Appendix B).

4. *β_0_* is the intercept, *β_1_, β_2_, β_3_* are the fixed effects coefficients; *u_0j_* is the random intercept and *u_1j_* and *u_2j_* are the random slopes for the receiver *j; ε_ij_* is the residual error term.

5. We acknowledge that there is more to competence than one’s score on the standardized aptitude test. Equally, charitable behavior is just one of many signs of someone being high on the warmth dimension. However, the intention of our study was to determine whether people rely on this limited information about other persons’ characteristics in making decisions in a new context (absent any additional task-relevant information about another person).

## References

[bibr1-01461672241301116] Abele A. E. (2022). Agency and communion as fundamental dimensions of social judgment–and Bogdan Wojciszke’s brilliant idea on perspective. Social Psychological Bulletin, 17, 1–8.

[bibr2-01461672241301116] Abele A. E. Cuddy A. J. Judd C. M. Yzerbyt V. Y. (2008). Fundamental dimensions of social judgment. European Journal of Social Psychology, 38(7), 1063–1065.

[bibr3-01461672241301116] Abele A. E. Ellemers N. Fiske S. T. Koch A. Yzerbyt V. (2021). Navigating the social world: Toward an integrated framework for evaluating self, individuals, and groups. Psychological Review, 128(2), 290–314. 32940512 10.1037/rev0000262

[bibr4-01461672241301116] Abele A. E. Wojciszke B. (2007). Agency and communion from the perspective of self versus others. Journal of Personality and Social Psychology, 93(5), 751–763. 17983298 10.1037/0022-3514.93.5.751

[bibr5-01461672241301116] Abele A. E. Wojciszke B. (2014). Communal and agentic content in social cognition: A dual perspective model. Advances in Experimental Social Psychology, 50, 195–255.

[bibr6-01461672241301116] Abeler J. Nosenzo D. Raymond C. (2019). Preferences for truth-telling. Econometrica, 87(4), 1115–1153.

[bibr7-01461672241301116] Akerlof G. A. (1970). The market for “lemons”: Quality uncertainty and the market mechanism. The Quarterly Journal of Economics, 84(3), 488–500.

[bibr8-01461672241301116] Alaoui L. Penta A. (2016). Endogenous depth of reasoning. The Review of Economic Studies, 83(4), 1297–1333.

[bibr9-01461672241301116] Albert M. Güth W. Kirchler E. Maciejovsky B. (2007). Are we nice(r) to nice(r) people? An experimental analysis. Experimental Economics, 10, 53–69.

[bibr10-01461672241301116] André J.-B. (2010). The evolution of reciprocity: Social types or social incentives? The American Naturalist, 175(2), 197–210. 10.1086/649597 20014939

[bibr11-01461672241301116] Apicella C. L. (2014). Upper-body strength predicts hunting reputation and reproductive success in Hadza hunter–gatherers. Evolution and Human Behavior, 35(6), 508–518.

[bibr12-01461672241301116] Apicella C. L. Marlowe F. W. Fowler J. H. Christakis N. A. (2012). Social networks and cooperation in hunter-gatherers. Nature, 481(7382), 497–501. 22281599 10.1038/nature10736 PMC3340565

[bibr13-01461672241301116] Arrow K. J. (1963). Uncertainty and the welfare economics of medical care. American Economic Review, 53(5), 941–973.

[bibr14-01461672241301116] Barclay P. (2006). Reputational benefits for altruistic punishment. Evolution and Human Behavior, 27(5), 325–344.

[bibr15-01461672241301116] Barclay P. (2013). Strategies for cooperation in biological markets, especially for humans. Evolution and Human Behavior, 34(3), 164–175.

[bibr16-01461672241301116] Barclay P. (2015). Reputation. In Buss D. (Ed.), The handbook of evolutionary psychology (2nd ed. pp. 1–19). Wiley.

[bibr17-01461672241301116] Barclay P. (2016). Biological markets and the effects of partner choice on cooperation and friendship. Current Opinion in Psychology, 7, 33–38.

[bibr18-01461672241301116] Barclay P. Reeve H. K. (2012). The varying relationship between helping and individual quality. Behavioral Ecology, 23(4), 693–698.

[bibr19-01461672241301116] Barclay P. Van Vugt M. (2015). The evolutionary psychology of human prosociality: Adaptations, byproducts, and mistakes. Schroeder D. A. Graziano W. G. (Eds.), The Oxford handbook of prosocial behavior (pp. 37–60). Oxford University Press.

[bibr20-01461672241301116] Barclay P. Willer R. (2007). Partner choice creates competitive altruism in humans. Proceedings of the Royal Society B: Biological Sciences, 274(1610), 749–753. 10.1098/rspb.2006.0209 PMC2197220 17255001

[bibr21-01461672241301116] Barker J. L. Power E. A. Heap S. Puurtinen M. Sosis R. (2019). Content, cost, and context: A framework for understanding human signaling systems. Evolutionary Anthropology: Issues, News, and Reviews, 28(2), 86–99. 10.1002/evan.21768 30869833

[bibr22-01461672241301116] Bates D. Kliegl R. Vasishth S. Baayen H. (2015). Parsimonious mixed models. arXiv:150604967.

[bibr23-01461672241301116] Baum J. Rabovsky M. Rose S. B. Abdel Rahman R. (2020). Clear judgments based on unclear evidence: Person evaluation is strongly influenced by untrustworthy gossip. Emotion, 20(2), 248–260. 30589302 10.1037/emo0000545

[bibr24-01461672241301116] Berg J. Dickhaut J. McCabe K. (1995). Trust, reciprocity, and social history. Games and Economic Behavior, 10(1), 122–142.

[bibr25-01461672241301116] Bolton G. E. Katok E. Ockenfels A. (2005). Cooperation among strangers with limited information about reputation. Journal of Public Economics, 89(8), 1457–1468.

[bibr26-01461672241301116] Brambilla M. Leach C. W. (2014). On the importance of being moral: The distinctive role of morality in social judgment. Social Cognition, 32(4), 397–408.

[bibr27-01461672241301116] Brambilla M. Rusconi P. Sacchi S. Cherubini P. (2011). Looking for honesty: The primary role of morality (vs. sociability and competence) in information gathering. European Journal of Social Psychology, 41(2), 135–143.

[bibr28-01461672241301116] Brambilla M. Sacchi S. Rusconi P. Goodwin G. P. (2021). The primacy of morality in impression development: Theory, research, and future directions. Advances in Experimental Social Psychology, 64, 187–262.

[bibr29-01461672241301116] Brandt H. Hauert C. Sigmund K. (2003). Punishment and reputation in spatial public goods games. Proceedings of the Royal Society of London. Series B: Biological Sciences, 270(1519), 1099–1104. 10.1098/rspb.2003.2336 PMC1691345 12803901

[bibr30-01461672241301116] Brandt H. Sigmund K. (2004). The logic of reprobation: Assessment and action rules for indirect reciprocation. Journal of Theoretical Biology, 231(4), 475–486. 15488525 10.1016/j.jtbi.2004.06.032

[bibr31-01461672241301116] Burks S. V. Carpenter J. P. Goette L. Rustichini A. (2009). Cognitive skills affect economic preferences, strategic behavior, and job attachment. Proceedings of the National Academy of Sciences, 106(19), 7745–7750. 10.1073/pnas.0812360106 PMC2683075 19416865

[bibr32-01461672241301116] Cacioppo J. T. Gardner W. L. Berntson G. G. (1997). Beyond bipolar conceptualizations and measures: The case of attitudes and evaluative space. Personality and Social Psychology Review, 1(1), 3–25. 15647126 10.1207/s15327957pspr0101_2

[bibr33-01461672241301116] Chen F. Guo T. (2021). Effects of competence information on perceptions of warmth. Asian Journal of Social Psychology, 24(4), 524–536.

[bibr34-01461672241301116] Cohen J. (1988). Statistical power analysis for the behavioral sciences (2nd ed.). Routledge.

[bibr35-01461672241301116] Cousineau D. O’Brien F. (2014). Error bars in within-subject designs: A comment on Baguley (2012). Behavior Research Methods, 46, 1149–1151. 24477859 10.3758/s13428-013-0441-z

[bibr36-01461672241301116] Cuddy A. Fiske S. Glick P. (2008). Competence and warmth as universal trait dimensions of interpersonal and intergroup perception: The stereotype content model and the bias map. Advances in Experimental Social Psychology, 40, 61–149.

[bibr37-01461672241301116] Dores Cruz T. D. Nieper A. S. Testori M. Martinescu E. Beersma B . (2021). An integrative definition and framework to study gossip. Group & Organization Management, 46(2), 252–285.

[bibr38-01461672241301116] Dores Cruz T. D. Thielmann I. Columbus S. Molho C. Wu J. Righetti F. De Vries R. E. Koutsoumpis A. Van Lange P. A. Beersma B. Balliet D . (2021). Gossip and reputation in everyday life. Philosophical Transactions of the Royal Society B, 376(1838), 20200301. 10.1098/rstb.2020.0301 PMC8487731 34601907

[bibr39-01461672241301116] Dores Cruz T. D. van der Lee R. Beersma B . (2021). Gossip about coronavirus: Infection status and norm adherence shape social responses. Group Processes & Intergroup Relations, 24(4), 658–679.

[bibr40-01461672241301116] Doutrelant C. McGregor P. (2000). Eavesdropping and mate choice in female fighting fish. Behaviour, 137(12), 1655–1668.

[bibr41-01461672241301116] Eisenbruch A. B. Krasnow M. M. (2022). Why warmth matters more than competence: A new evolutionary approach. Perspectives on Psychological Science, 17(6), 1604–1623. 35748187 10.1177/17456916211071087

[bibr42-01461672241301116] Emler N. (2019). Human sociality and psychological foundations. In Giardini F. Wittek R. (Eds.), The Oxford handbook of gossip and reputation (pp. 47–68). Oxford University Press.

[bibr43-01461672241301116] Fiske S. T. (1980). Attention and weight in person perception: The impact of negative and extreme behavior. Journal of Personality and Social Psychology, 38(6), 889–906.

[bibr44-01461672241301116] Fiske S. T. (1993). Social cognition and social perception. Annual Review of Psychology, 44(1), 155–194. 10.1146/annurev.ps.44.020193.001103 8434891

[bibr45-01461672241301116] Fiske S. T. (2018). Stereotype content: Warmth and competence endure. Current Directions in Psychological Science, 27(2), 67–73. 29755213 10.1177/0963721417738825 PMC5945217

[bibr46-01461672241301116] Fiske S. T. Cuddy A. J. Glick P. (2007). Universal dimensions of social cognition: Warmth and competence. Trends in Cognitive Sciences, 11(2), 77–83. 17188552 10.1016/j.tics.2006.11.005

[bibr47-01461672241301116] Fonseca M. A. Peters K. (2018). Will any gossip do? Gossip does not need to be perfectly accurate to promote trust. Games and Economic Behavior, 107, 253–281.

[bibr48-01461672241301116] Garfield Z. H. Schacht R. Post E. R. Ingram D. Uehling A. Macfarlan S. J. (2021). The content and structure of reputation domains across human societies: A view from the evolutionary social sciences. Philosophical Transactions of the Royal Society B, 376(1838), 20200296. 10.1098/rstb.2020.0296 PMC8487742 34601916

[bibr49-01461672241301116] Garfield Z. H. von Rueden C. Hagen E. H. (2019). The evolutionary anthropology of political leadership. The Leadership Quarterly, 30(1), 59–80.

[bibr50-01461672241301116] Giardini F. Balliet D. Power E. A. Számadó S. Takács K. (2022). Four puzzles of reputation-based cooperation: Content, process, honesty, and structure. Human Nature, 33(1), 43–61. 34961914 10.1007/s12110-021-09419-3 PMC8964644

[bibr51-01461672241301116] Gill D. Prowse V. (2016). Cognitive ability, character skills, and learning to play equilibrium: A level-k analysis. Journal of Political Economy, 124(6), 1619–1676.

[bibr52-01461672241301116] Goodwin G. P. (2015). Moral character in person perception. Current Directions in Psychological Science, 24(1), 38–44.

[bibr53-01461672241301116] Goodwin G. P. Piazza J. Rozin P. (2014). Moral character predominates in person perception and evaluation. Journal of Personality and Social Psychology, 106(1), 148–168. 24274087 10.1037/a0034726

[bibr54-01461672241301116] Gräf M. Unkelbach C. (2016). Halo effects in trait assessment depend on information valence: Why being honest makes you industrious, but lying does not make you lazy. Personality and Social Psychology Bulletin, 42(3), 290–310. 26811437 10.1177/0146167215627137

[bibr55-01461672241301116] Grafen A. (1990). Biological signals as handicaps. Journal of Theoretical Biology, 144(4), 517–546. 2402153 10.1016/s0022-5193(05)80088-8

[bibr56-01461672241301116] Gurven M. Allen-Arave W. Hill K. Hurtado A. M. (2001). Reservation food sharing among the ache of Paraguay. Human Nature, 12, 273–297. 26192409 10.1007/s12110-001-1000-3

[bibr57-01461672241301116] Gurven M. Allen-Arave W. Hill K. Hurtado A. M. (2000). “It’s a wonderful life”: Signaling generosity among the Ache of Paraguay. Evolution and Human Behavior, 21(4), 263–282. 10.1016/s1090-5138(00)00032-5 10899478

[bibr58-01461672241301116] Hackel L. M. Doll B. B. Amodio D. M. (2015). Instrumental learning of traits versus rewards: Dissociable neural correlates and effects on choice. Nature Neuroscience, 18(9), 1233–1235. 26237363 10.1038/nn.4080

[bibr59-01461672241301116] Hackel L. M. Mende-Siedlecki P. Loken S. Amodio D. M. (2022). Context-dependent learning in social interaction: Trait impressions support flexible social choices. Journal of Personality and Social Psychology, 123(4), 655–675. 35113628 10.1037/pspa0000296

[bibr60-01461672241301116] Hackel L. M. Zaki J. (2018). Propagation of economic inequality through reciprocity and reputation. Psychological Science, 29(4), 604–613. 29474134 10.1177/0956797617741720

[bibr61-01461672241301116] Hauke N. Abele A. E. (2020). The impact of negative gossip on target and receiver. A “big two” analysis. Basic and Applied Social Psychology, 42(2), 115–132.

[bibr62-01461672241301116] Hayashi N. Yamagishi T. (1998). Selective play: Choosing partners in an uncertain world. Personality and Social Psychology Review, 2(4), 276–289. 15647134 10.1207/s15327957pspr0204_4

[bibr63-01461672241301116] Heck P. R. Krueger J. I. (2017). Social perception in the volunteer’s dilemma: Role of choice, outcome, and expectation. Social Cognition, 35(5), 497–519.

[bibr64-01461672241301116] Henrich J. Gil-White F. J. (2001). The evolution of prestige: Freely conferred deference as a mechanism for enhancing the benefits of cultural transmission. Evolution and Human Behavior, 22(3), 165–196. 10.1016/s1090-5138(00)00071-4 11384884

[bibr65-01461672241301116] Henrich J. Heine S. J. Norenzayan A. (2010). The weirdest people in the world? Behavioral and Brain Sciences, 33(2–3), 61–83. 20550733 10.1017/S0140525X0999152X

[bibr66-01461672241301116] Hess N. H. Hagen E. H. (2021). Competitive gossip: The impact of domain, resource value, resource scarcity and coalitions. Philosophical Transactions of the Royal Society B, 376(1838), 20200305. 10.1098/rstb.2020.0305 PMC8487733 34601911

[bibr67-01461672241301116] Jordan J. J. Homan M. Nowak M. A. Rand D. G. (2016). Uncalculating cooperation is used to signal trustworthiness. Proceedings of the National Academy of Sciences, 113(31), 8658–8663. 10.1073/pnas.1601280113 PMC4978259 27439873

[bibr68-01461672241301116] Judd C. M. James-Hawkins L. Yzerbyt V. Kashima Y. (2005). Fundamental dimensions of social judgment: Understanding the relations between judgments of competence and warmth. Journal of Personality and Social Psychology, 89(6), 899–913. 16393023 10.1037/0022-3514.89.6.899

[bibr69-01461672241301116] Koch A. Yzerbyt V. Abele A. Ellemers N. Fiske S. T. (2021). Social evaluation: Comparing models across interpersonal, intragroup, intergroup, several-group, and many-group contexts. In Gawronski B. (Ed.), Advances in experimental social psychology (Vol. 63, pp. 1–68). Elsevier.

[bibr70-01461672241301116] Krueger J. I. (2019). The vexing volunteer’s dilemma. Current Directions in Psychological Science, 28(1), 53–58.

[bibr71-01461672241301116] Krueger J. I. Acevedo M. (2007). Perceptions of self and other in the prisoner’s dilemma: Outcome bias and evidential reasoning. The American Journal of Psychology, 120(4), 593–618. 18277518

[bibr72-01461672241301116] Krueger J. I. Heck P. R. Evans A. M. DiDonato T. E. (2020). Social game theory: Preferences, perceptions, and choices. European Review of Social Psychology, 31(1), 222–253.

[bibr73-01461672241301116] Kummer H. (1978). On the value of social relationships to nonhuman primates: A heuristic scheme. Social Science Information, 17(4–5), 687–705.

[bibr74-01461672241301116] Lambrecht M. Proto E. Rustichini A. Sofianos A. (2024). Intelligence disclosure and cooperation in repeated interactions. American Economic Journal: Microeconomics, 16(3), 199–231.

[bibr75-01461672241301116] Luttrell A. Sacchi S. Brambilla M. (2022). Changing impressions in competence-oriented domains: The primacy of morality endures. Journal of Experimental Social Psychology, 98, 104246.

[bibr76-01461672241301116] Lyle H. F. III Smith E. A. (2014). The reputational and social network benefits of prosociality in an Andean community. Proceedings of the National Academy of Sciences, 111(13), 4820–4825. 10.1073/pnas.1318372111 PMC3977260 24639494

[bibr77-01461672241301116] Macfarlan S. J. Lyle H. F. (2015). Multiple reputation domains and cooperative behaviour in two Latin American communities. Philosophical Transactions of the Royal Society B: Biological Sciences, 370(1683), 20150009. 10.1098/rstb.2015.0009 PMC4633845 26503682

[bibr78-01461672241301116] Macfarlan S. J. Quinlan R. Remiker M. (2013). Cooperative behaviour and prosocial reputation dynamics in a Dominican village. Proceedings of the Royal Society B: Biological Sciences, 280(1761), 20130557. 10.1098/rspb.2013.0557 PMC3652444 23760642

[bibr79-01461672241301116] Mattan B. D. Kubota J. T. Cloutier J. (2017). How social status shapes person perception and evaluation: A social neuroscience perspective. Perspectives on Psychological Science, 12(3), 468–507. 28544863 10.1177/1745691616677828

[bibr80-01461672241301116] McGregor P. K. Peake T. M. (2000). Communication networks: Social environments for receiving and signalling behaviour. Acta Ethologica, 2, 71–81.

[bibr81-01461672241301116] McNamara J. M. Barta Z. Fromhage L. Houston A. I. (2008). The coevolution of choosiness and cooperation. Nature, 451(7175), 189–192. 18185587 10.1038/nature06455

[bibr82-01461672241301116] Melis A. P. Hare B. Tomasello M. (2006). Chimpanzees recruit the best collaborators. Science, 311(5765), 1297–1300. 16513985 10.1126/science.1123007

[bibr83-01461672241301116] Mesoudi A. Whiten A. Dunbar R. (2006). A bias for social information in human cultural transmission. British Journal of Psychology, 97(3), 405–423. 16848951 10.1348/000712605X85871

[bibr84-01461672241301116] Milinski M. (2019). Gossip and reputation in social dilemmas. In Giardini F. Wittek R. (Eds.), The Oxford handbook of gossip and reputation (pp. 193–213). Oxford.

[bibr85-01461672241301116] Milinski M. Semmann D. Krambeck H.-J. (2002). Reputation helps solve the “tragedy of the commons.” Nature, 415(6870), 424–426. 11807552 10.1038/415424a

[bibr86-01461672241301116] Molho C. Tybur J. M. Van Lange P. A. Balliet D. (2020). Direct and indirect punishment of norm violations in daily life. Nature Communications, 11(1), 3432. 10.1038/s41467-020-17286-2 PMC7347610 32647165

[bibr87-01461672241301116] Nisbett R. E. Borgida E. Crandall R. Reed H. (1977). Popular induction: Information is not necessarily informative. In Carroll J. S. Payne J. W. (Eds.), Cognition and social behavior (1st ed.), 113–133. Lawrence Erlbaum.

[bibr88-01461672241301116] Noë R. Hammerstein P. (1994). Biological markets: Supply and demand determine the effect of partner choice in cooperation, mutualism and mating. Behavioral Ecology and Sociobiology, 35, 1–11.

[bibr89-01461672241301116] Noë R. Hammerstein P. (1995). Biological markets. Trends in Ecology & Evolution, 10(8), 336–339. 21237061 10.1016/s0169-5347(00)89123-5

[bibr90-01461672241301116] Nowak M. A. (2006). Five rules for the evolution of cooperation. Science, 314(5805), 1560–1563. 17158317 10.1126/science.1133755 PMC3279745

[bibr91-01461672241301116] Nowak M. A. Sigmund K. (1998). Evolution of indirect reciprocity by image scoring. Nature, 393(6685), 573–577. 9634232 10.1038/31225

[bibr92-01461672241301116] Ohtsubo Y. Watanabe E. (2009). Do sincere apologies need to be costly? Test of a costly signaling model of apology. Evolution and Human Behavior, 30(2), 114–123.

[bibr93-01461672241301116] Ohtsuki H. Iwasa Y. (2004). How should we define goodness? Reputation dynamics in indirect reciprocity. Journal of Theoretical Biology, 231(1), 107–120. 15363933 10.1016/j.jtbi.2004.06.005

[bibr94-01461672241301116] Ohtsuki H. Iwasa Y. (2006). The leading eight: Social norms that can maintain cooperation by indirect reciprocity. Journal of Theoretical Biology, 239(4), 435–444. 16174521 10.1016/j.jtbi.2005.08.008

[bibr95-01461672241301116] Oliveira R. F. McGregor P. K. Latrue C. (1998). Know thine enemy: Fighting fish gather information from observing conspecific interactions. Proceedings of the Royal Society of London. Series B: Biological Sciences, 265(1401), 1045–1049.

[bibr96-01461672241301116] Ophir A. G. Persaud K. N. Galef B. G Jr . (2005). Avoidance of relatively aggressive male Japanese quail (coturnix japonica) by sexually experienced conspecific females. Journal of Comparative Psychology, 119(1), 3–7. 15740424 10.1037/0735-7036.119.1.3

[bibr97-01461672241301116] Otter K. McGregor P. K. Terry A. M. R. Burford F. R. Peake T. M. Dabelsteen T. (1999). Do female great tits (Parus Major) assess males by eavesdropping? A field study using interactive song playback. Proceedings of the Royal Society of London. Series B: Biological Sciences, 266(1426), 1305–1309.

[bibr98-01461672241301116] Otter K. A. Stewart I. R. McGregor P. K. Terry A. M. Dabelsteen T. Burke T. (2001). Extra-pair paternity among great tits parus major following manipulation of male signals. Journal of Avian Biology, 32(4), 338–344.

[bibr99-01461672241301116] Peeters G. (2002). From good and bad to can and must: Subjective necessity of acts associated with positively and negatively valued stimuli. European Journal of Social Psychology, 32(1), 125–136.

[bibr100-01461672241301116] Peeters G. (2008). The evaluative face of a descriptive model: Communion and agency in Peabody’s tetradic model of trait organization. European Journal of Social Psychology, 38(7), 1066–1072.

[bibr101-01461672241301116] Peters K. Kashima Y. (2015). Bad habit or social good? How perceptions of gossiper morality are related to gossip content. European Journal of Social Psychology, 45(6), 784–798.

[bibr102-01461672241301116] Proto E. Rustichini A. Sofianos A. (2019). Intelligence, personality, and gains from cooperation in repeated interactions. Journal of Political Economy, 127(3), 1351–1390.

[bibr103-01461672241301116] Reeder G. D. Kumar S. Hesson-McInnis M. S. Trafimow D. (2002). Inferences about the morality of an aggressor: The role of perceived motive. Journal of Personality and Social Psychology, 83(4), 789–803. 12374435 10.1037//0022-3514.83.4.789

[bibr104-01461672241301116] Roberts G. (2015). Human cooperation: The race to give. Current Biology, 25(10), R425–R427. 10.1016/j.cub.2015.03.045 25989085

[bibr105-01461672241301116] Romano A. Giardini F. Columbus S. de Kwaadsteniet E. W. Kisfalusi D. Triki Z. Snijders C. Hagel K. (2021). Reputation and socio-ecology in humans. Philosophical Transactions of the Royal Society B, 376(1838), 20200295. 10.1098/rstb.2020.0295 PMC8487743 34601915

[bibr106-01461672241301116] Rosenberg S. Nelson C. Vivekananthan P. (1968). A multidimensional approach to the structure of personality impressions. Journal of Personality and Social Psychology, 9(4), 283–294. 5670821 10.1037/h0026086

[bibr107-01461672241301116] Seinen I. Schram A. (2006). Social status and group norms: Indirect reciprocity in a repeated helping experiment. European Economic Review, 50(3), 581–602.

[bibr108-01461672241301116] Singmann H. Kellen D. (2019). An introduction to mixed models for experimental psychology. In Spieler D. H. Schumacher E. (Eds.), New methods in cognitive psychology (pp. 4–31). Routledge.

[bibr109-01461672241301116] Smith E. A. (2004). Why do good hunters have higher reproductive success? Human Nature, 15, 343–364. 26189411 10.1007/s12110-004-1013-9

[bibr110-01461672241301116] Smith E. A. Bird R. B. (2005). Costly signaling and cooperative behavior. In Gintis H. Bowles S. Boyd R. Fehr E. (Eds.), Moral sentiments and material interests (pp. 115–148). MIT Press.

[bibr111-01461672241301116] Smith K. M. Apicella C. L. (2020). Partner choice in human evolution: The role of cooperation, foraging ability, and culture in Hadza campmate preferences. Evolution and Human Behavior, 41(5), 354–366.

[bibr112-01461672241301116] Smith K. M. Mabulla I. A. Apicella C. L. (2022). Hadza hunter–gatherers with greater exposure to other cultures share more with generous campmates. Biology Letters, 18(7), 20220157. 35857893 10.1098/rsbl.2022.0157 PMC9277280

[bibr113-01461672241301116] Sommerfeld R. D. Krambeck H.-J. Semmann D. Milinski M. (2007). Gossip as an alternative for direct observation in games of indirect reciprocity. Proceedings of the National Academy of Sciences, 104(44), 17435–17440. 10.1073/pnas.0704598104 PMC2077274 17947384

[bibr114-01461672241301116] Spence M. (1974). Competitive and optimal responses to signals: An analysis of efficiency and distribution. Journal of Economic Theory, 7(3), 296–332.

[bibr115-01461672241301116] Stellar J. E. Willer R. (2018). Unethical and inept? The influence of moral information on perceptions of competence. Journal of Personality and Social Psychology, 114(2), 195–210. 29389214 10.1037/pspa0000097

[bibr116-01461672241301116] Thorndike E. L. (1920). A constant error in psychological ratings. Journal of Applied Psychology, 4(1), 25–29.

[bibr117-01461672241301116] Tognetti A. Berticat C. Raymond M. Faurie C. (2014). Assortative mating based on cooperativeness and generosity. Journal of Evolutionary Biology, 27(5), 975–981. 24581285 10.1111/jeb.12346

[bibr118-01461672241301116] Tooby J. Cosmides L. (1996). Friendship and the banker’s paradox: Other pathways to the evolution of adaptations for altruism. Proceedings of the British Academy, 88, 119–144.

[bibr119-01461672241301116] Vail A. L. Manica A. Bshary R. (2014). Fish choose appropriately when and with whom to collaborate. Current Biology, 24(17), R791–R793. 10.1016/j.cub.2014.07.033 25202866

[bibr120-01461672241301116] van Dijk E. De Dreu C. K . (2021). Experimental games and social decision making. Annual Review of Psychology, 72, 415–438. 10.1146/annurev-psych-081420-110718 33006926

[bibr121-01461672241301116] Van Vugt M. Roberts G. Hardy C . (2007). Competitive altruism: Development of reputation-based cooperation in groups. In Dunbar R. Barrett L. (Eds.), Handbook of evolutionary psychology (pp. 531–540). Oxford University Press.

[bibr122-01461672241301116] Von Rueden C. Gurven M. Kaplan H. Stieglitz J . (2014). Leadership in an egalitarian society. Human Nature, 25, 538–566. 25240393 10.1007/s12110-014-9213-4 PMC4258461

[bibr123-01461672241301116] Wedekind C. Braithwaite V. A. (2002). The long-term benefits of human generosity in indirect reciprocity. Current Biology, 12(12), 1012–1015. 12123575 10.1016/s0960-9822(02)00890-4

[bibr124-01461672241301116] Wojciszke B. (1994). Multiple meanings of behavior: Construing actions in terms of competence or morality. Journal of Personality and Social Psychology, 67(2), 222–232.

[bibr125-01461672241301116] Wojciszke B. (2005). Morality and competence in person-and self-perception. European Review of Social Psychology, 16(1), 155–188.

[bibr126-01461672241301116] Wojciszke B. Abele A. E. (2018). Agency and communion in social cognition. In Abele A. E. Wojciszke B. (Eds.), Agency and communion in social psychology (pp. 25–38). Routledge.

[bibr127-01461672241301116] Wojciszke B. Bazinska R. Jaworski M. (1998). On the dominance of moral categories in impression formation. Personality and Social Psychology Bulletin, 24(12), 1251–1263.

[bibr128-01461672241301116] Wojciszke B. Brycz H. Borkenau P. (1993). Effects of information content and evaluative extremity on positivity and negativity biases. Journal of Personality and Social Psychology, 64(3), 327–335.

[bibr129-01461672241301116] Wu J. Balliet D. Van Lange P. A. (2016). Reputation, gossip, and human cooperation. Social and Personality Psychology Compass, 10(6), 350–364.

[bibr130-01461672241301116] Yao B. Scott G. G. McAleer P. O’Donnell P. J. Sereno S. C. (2014). Familiarity with interest breeds gossip: Contributions of emotion, expectation, and reputation. PLOS ONE, 9(8), Article e104916. 10.1371/journal.pone.0104916 PMC4132070 25119267

[bibr131-01461672241301116] Zahavi A. (1977). The cost of honesty (further remarks on the handicap principle). Journal of Theoretical Biology, 67(3), 603–605. 904334 10.1016/0022-5193(77)90061-3

[bibr132-01461672241301116] Zahavi A. Zahavi A. (1997). The handicap principle: A missing piece of Darwin’s puzzle. Oxford University Press.

