# Supplementary material for: Multiple Reputations: Selective Attention to Competence and Character
Source: Pers Soc Psychol Bull. 2024 Dec 6;52(4):896–914. doi: 10.1177/01461672241301116 (PMC12949748; doi:10.1177/01461672241301116)
Supplement: sj-docx-1-psp-10.1177_01461672241301116 – Supplemental material for Multiple Reputations: Selective Attention to Competence and Character [file sj-docx-1-psp-10.1177_01461672241301116.docx]

**Appendix A**

**Primary Analysis**

Following non-convergence of our maximal models, we first dropped the correlations between random effects from the model. However, in all three experiments, this model specification still produced a singular fit, so we next dropped the random effects for the interaction term. This led to successfully converging models, which were reported in the main manuscript. Here we report the results for all three specifications of the mixed models separately for Experiment 1 in Table A1, for Experiment 2 in Table A2 and for Experiment 3 in Table A3.

The regression equation for our final model of Experiments 1 and 3 is: *Sender’s Investment Amount_ij_ = β_0_ + β_1_ Receiver's Helpfulness_ij_ + β_2_Receiver's Competence_ij_ + β_3_(Receiver's Helpfulness_ij_ Receiver's Competence_ij_) + u_0j_ + u_1j_ Receiver's Helpfulness_ij_ + u_2j_Receiver's Competence_ij_ + ε_ij_*^[[1]](#footnote-1)^.

For Experiment 2, the regression equation for our final model is: *Sender’s Investment Amount_ij_ = β_0_ + β_1_ Receiver's Helpfulness_ij_ + β_2_Receiver's Competence_ij_ + β_3_(Receiver's Helpfulness_ij_ Receiver's Competence_ij_) + u_0j_ + u_1j_ Receiver's Helpfulness_ij_ + ε_ij_*^[[2]](#footnote-2)^.

Crucially, across all model specifications, we found that the pattern of results was qualitatively very similar, with only slight variations in the obtained coefficients. This is further displayed through the plots and pairwise comparison tests.

| **Table A1.**  Experiment 1 Primary Mixed Model Results | | | | |
| --- | --- | --- | --- | --- |
| **Mixed Model Anova Table (Type 3 tests, Satterthwaite-method)** | **df** | **F** | ***_η_^2^_p_*** | **p** |
| *Maximal Model (by-participant slopes and intercepts, plus the correlations among them)* | | | | |
| Receiver’s Helpfulness Reputation | 2, 100 | 80.74 | .62 | <.001 |
| Receiver’s Competence Reputation | 2, 103 | 25.87 | .32 | <.001 |
| Receiver’s Helpfulness Reputation * Receiver’s Competence Reputation | 4, 216 | 5.55 | .09 | <.001 |
| *Reduced Model (by-participant slopes and intercepts, dropping the correlations between random effects)* | | | | |
| Receiver’s Helpfulness Reputation | 2, 100 | 77.15 | .61 | <.001 |
| Receiver’s Competence Reputation | 2, 102 | 25.63 | .33 | <.001 |
| Receiver’s Helpfulness Reputation * Receiver’s Competence Reputation | 4, 564 | 6.09 | .04 | <.001 |
| *Reduced Model (by-participant slopes and intercepts, dropping the correlations between random effects and dropped the random effects for the interaction term)* | | | | |
| Receiver’s Helpfulness Reputation | 2, 100 | 77.24 | .61 | <.001 |
| Receiver’s Competence Reputation | 2, 102 | 25.61 | .33 | <.001 |
| Receiver’s Helpfulness Reputation * Receiver’s Competence Reputation | 4, 1317 | 6.86 | .02 | <.001 |
|  | | | | |

| **Table A2.**  Experiment 2 Primary Mixed Model Results | | | | |
| --- | --- | --- | --- | --- |
| **Mixed Model Anova Table (Type 3 tests, Satterthwaite-method)** | **df** | **F** | ***_η_^2^_p_*** | **p** |
| *Maximal Model (by-participant slopes and intercepts, plus the correlations among them)* | | | | |
| Receiver’s Helpfulness Reputation | 2, 251 | 214.48 | .63 | <.001 |
| Receiver’s Competence Reputation | 2, 363 | 45.63 | .20 | <.001 |
| Receiver’s Helpfulness Reputation * Receiver’s Competence Reputation | 4, 466 | 15.04 | .11 | <.001 |
| *Reduced Model (by-participant slopes and intercepts, dropping the correlations between random effects)* | | | | |
| Receiver’s Helpfulness Reputation | 2, 254 | 213.83 | .63 | <.001 |
| Receiver’s Competence Reputation | 2, 501 | 45.07 | .15 | <.001 |
| Receiver’s Helpfulness Reputation * Receiver’s Competence Reputation | 4, 1242 | 14.54 | .04 | <.001 |
| *Reduced Model (by-participant slopes and intercepts, dropping the correlations between random effects and dropped the random effects for the interaction term)* | | | | |
| Receiver’s Helpfulness Reputation | 2, 254 | 214.62 | .63 | <.001 |
| Receiver’s Competence Reputation | 2, 3779 | 143.19 | .07 | <.001 |
| Receiver’s Helpfulness Reputation * Receiver’s Competence Reputation | 4, 3779 | 16.27 | .02 | <.001 |
|  | | | | |

| **Table A3.**  Experiment 3 Primary Mixed Model Results | | | | |
| --- | --- | --- | --- | --- |
| **Mixed Model Anova Table (Type 3 tests, Satterthwaite-method)** | **df** | **F** | ***_η_^2^_p_*** | **p** |
| *Maximal Model (by-participant slopes and intercepts, plus the correlations among them)* | | | | |
| Receiver’s Helpfulness Reputation | 2, 285 | 102.50 | .42 | <.001 |
| Receiver’s Competence Reputation | 2, 253 | 314.33 | .71 | <.001 |
| Receiver’s Helpfulness Reputation * Receiver’s Competence Reputation | 4, 418 | 20.05 | .16 | <.001 |
| *Reduced Model (by-participant slopes and intercepts, dropping the correlations between random effects)* | | | | |
| Receiver’s Helpfulness Reputation | 2, 260 | 102.80 | .44 | <.001 |
| Receiver’s Competence Reputation | 2, 256 | 335.03 | .72 | <.001 |
| Receiver’s Helpfulness Reputation * Receiver’s Competence Reputation | 4, 459 | 22.25 | .16 | <.001 |
| *Reduced Model (by-participant slopes and intercepts, dropping the correlations between random effects and dropped the random effects for the interaction term)* | | | | |
| Receiver’s Helpfulness Reputation | 2, 262 | 103.33 | .44 | <.001 |
| Receiver’s Competence Reputation | 2, 256 | 335.32 | .72 | <.001 |
| Receiver’s Helpfulness Reputation * Receiver’s Competence Reputation | 4, 3289 | 32.35 | .04 | <.001 |
|  | | | | |

We additionally report the results follow-up pairwise comparison tests, where receivers’ helpfulness (here the diagnostic cue) is the predictor and receivers’ competence is the moderator, for Experiment 1 in Table A4 (and Figure A1) and for Experiment 2 in Table A5(and Figure A2). In Table A6 (and Figure A3) we report the results for our pairwise comparisons, of our analysis where receivers’ competence (here the diagnostic cue) is the predictor and receivers’ helpfulness is the moderator.

| **Table A4.**  Experiment 1 results for follow-up pairwise comparison tests | | | | | | |
| --- | --- | --- | --- | --- | --- | --- |
| **Receiver’s**  **Competence**  **Reputation** | **Receiver’s**  **Helpfulness**  **Reputation** | **Estimate** | **SE** | **df** | **z** | **p** |
| Low | Low - Medium | -4.77 | 0.86 | 462 | -5.53 | <.001 |
|  | Low - High | -11.81 | 1.26 | 149 | -9.35 | <.001 |
|  | Medium - High | -7.04 | .91 | 355 | -7.76 | <.001 |
| Medium | Low - Medium | -5.94 | .86 | 462 | -6.89 | <.001 |
|  | Low - High | -13.68 | 1.26 | 149 | -10.83 | <.001 |
|  | Medium - High | -7.74 | .91 | 355 | -8.53 | <.001 |
| High | Low - Medium | -6.67 | .86 | 462 | -7.73 | <.001 |
|  | Low - High | -16.58 | 1.26 | 149 | -13.13 | <.001 |
|  | Medium - High | -9.92 | .91 | 355 | -10.93 | <.001 |
| Dependent Variable: Senders’ Level of Investment  Degrees-of-freedom method: Kenward-Roger  P value adjustment: Bonferroni method for 3 tests  *Note*. The receiver’s helpfulness (here the diagnostic cue) is the predictor and receiver’s competence is the moderator | | | | | | |

**Figure A1.**

Results of Experiment 1


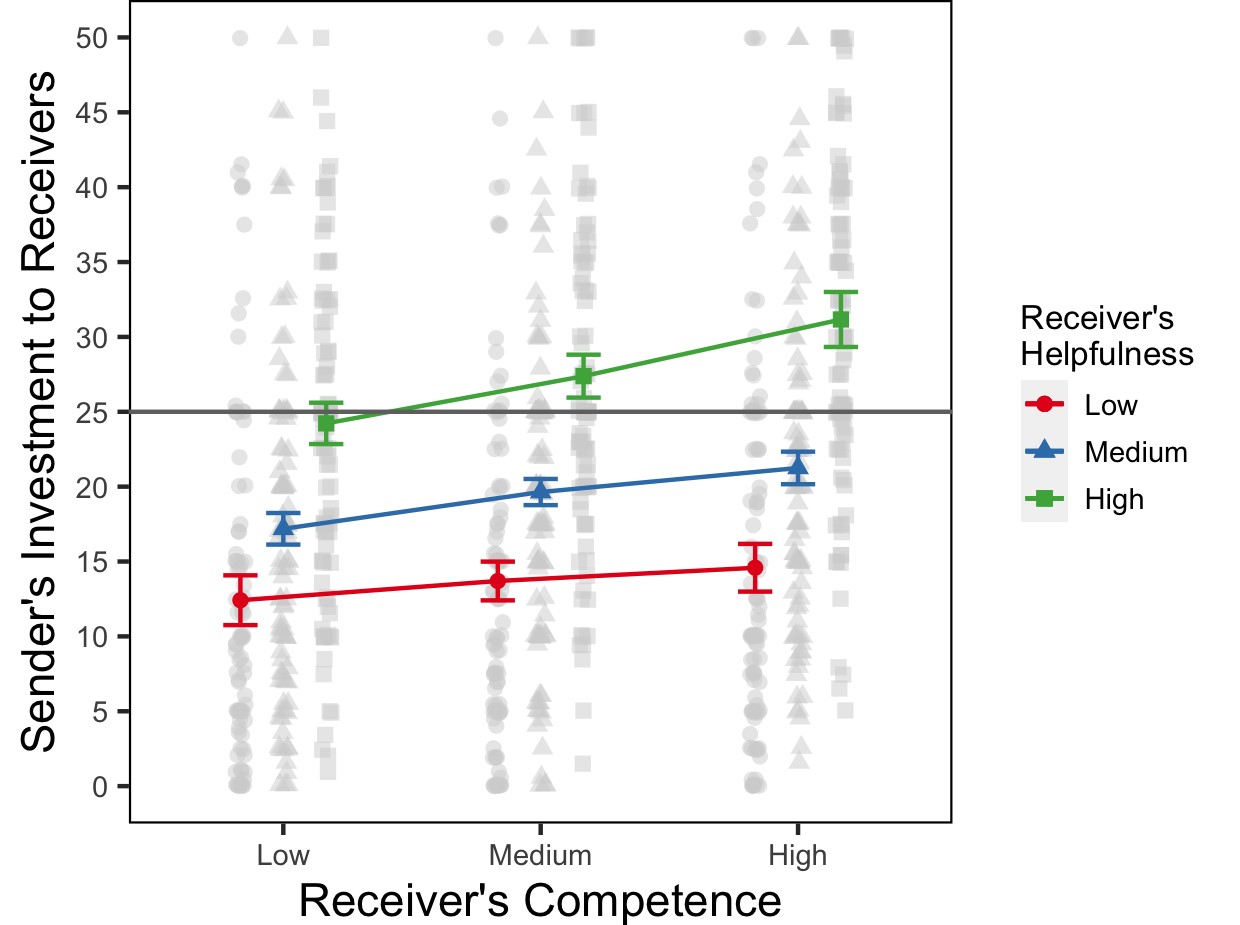


*Note.* Figure shows the marginal means for the amounts sent by senders to receivers from the random effects model. The figure includes within-subject (Cousineau & O’Brien, 2014) error bars of the senders’ level of investment to each receiver. The raw data of the senders’ level of investment is shown in gray. The gray horizontal line signifies half of the senders’ endowment. The x-axis shows three reputation levels representing the receivers’ competence (low, medium, high), the outcome-irrelevant cue in this experiment.

**Figure A2.**

Results of Experiment 2


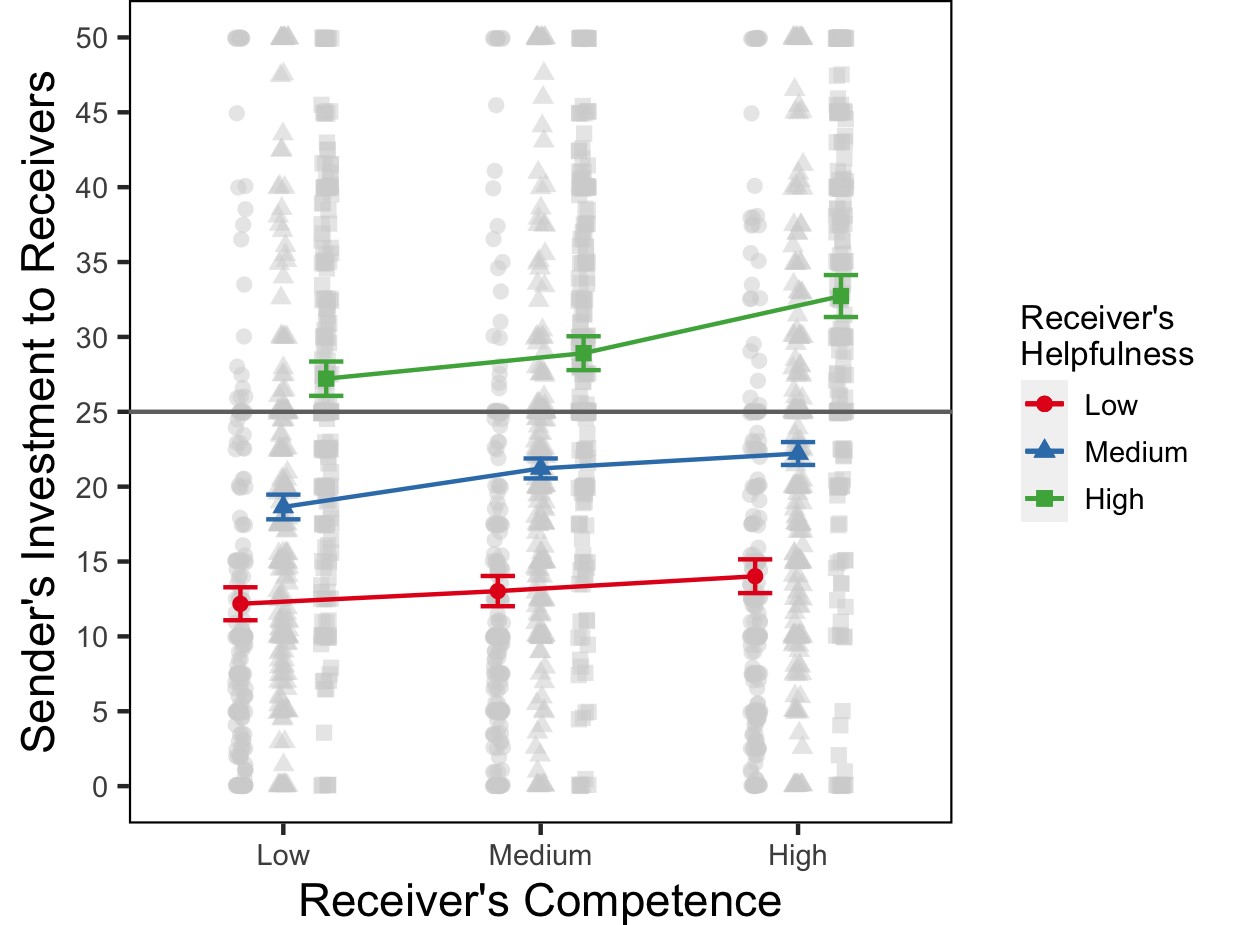


*Note.* Figure shows the marginal means for the amounts sent by senders to receivers from the random effects model. The figure includes within-subject (Cousineau & O’Brien, 2014) error bars of the senders’ level of investment to each receiver. The raw data of the senders’ level of investment is shown in gray. The gray horizontal line signifies half of the senders’ endowment. The x-axis shows three reputation levels representing the receivers’ competence (low, medium, high), the outcome-irrelevant cue in this experiment.

| **Table A5.**  Experiment 2 results for follow-up pairwise comparison tests | | | | | | |
| --- | --- | --- | --- | --- | --- | --- |
| **Receiver’s**  **Competence**  **Reputation** | **Receiver’s**  **Helpfulness**  **Reputation** | **Estimate** | **SE** | **df** | **z** | **p** |
| Low | Low - Medium | -6.46 | .56 | 891 | -11.65 | <.001 |
|  | Low - High | -15.03 | .86 | 346 | -17.56 | <.001 |
|  | Medium - High | -8.57 | .62 | 723 | -13.72 | <.001 |
| Medium | Low - Medium | -8.19 | .56 | 891 | -14.76 | <.001 |
|  | Low - High | -15.89 | .86 | 346 | -18.56 | <.001 |
|  | Medium - High | -7.70 | .62 | 723 | -12.33 | <.001 |
| High | Low - Medium | -8.19 | .56 | 891 | -14.76 | <.001 |
|  | Low - High | -18.71 | .86 | 346 | -21.86 | <.001 |
|  | Medium - High | -10.52 | .62 | 723 | -16.85 | <.001 |
| Dependent Variable: Senders’ Level of Investment  Degrees-of-freedom method: Kenward-Roger  P value adjustment: Bonferroni method for 3 tests  *Note.* The receiver’s helpfulness (here the diagnostic cue) is the predictor and receiver’s competence is the moderator | | | | | | |

**Figure A3.**

Results of Experiment 3


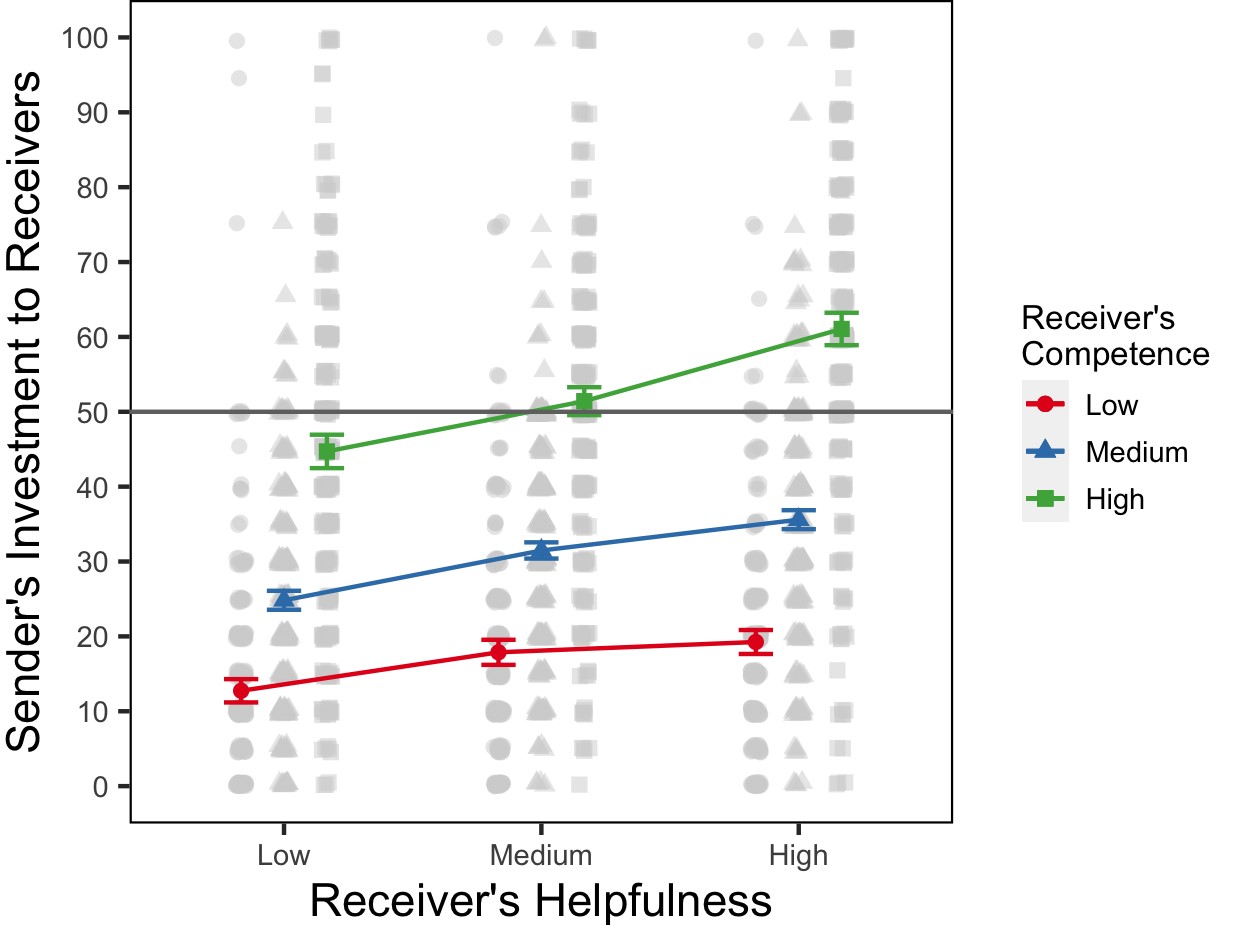


*Note.* Figure shows the marginal means for the amounts sent by senders to receivers from the random effects model. The figure includes within-subject (Cousineau & O’Brien, 2014) error bars of the senders’ level of investment to each receiver. The raw data of the senders’ level of investment is shown in gray. The gray horizontal line signifies half of the senders’ endowment. The x-axis shows three reputation levels representing the receivers’ helpfulness (low, medium, high), the outcome-irrelevant cue in this experiment.

| **Table A6.**  Experiment 3 results for follow-up pairwise comparison tests | | | | | | |
| --- | --- | --- | --- | --- | --- | --- |
| **Receiver’s**  **Helpfulness**  **Reputation** | **Receiver’s**  **Competence**  **Reputation** | **Estimate** | **SE** | **df** | **z** | **p** |
| Low | Low - Medium | -12.09 | .98 | 942 | -12.37 | <.001 |
|  | Low - High | -31.97 | 1.49 | 356 | -21.46 | <.001 |
|  | Medium - High | -19.88 | 1.11 | 725 | -17.92 | <.001 |
| Medium | Low - Medium | -13.60 | .98 | 942 | -13.92 | <.001 |
|  | Low - High | -33.54 | 1.49 | 356 | -22.52 | <.001 |
|  | Medium - High | -19.94 | 1.11 | 725 | -17.97 | <.001 |
| High | Low - Medium | -16.34 | .98 | 942 | -16.72 | <.001 |
|  | Low - High | -41.81 | 1.49 | 356 | -28.07 | <.001 |
|  | Medium - High | -25.47 | 1.11 | 725 | -22.95 | <.001 |
| Dependent Variable: Senders’ Level of Investment  Degrees-of-freedom method: Kenward-Roger  P value adjustment: Bonferroni method for 3 tests  *Note.* The receiver’s competence (here the diagnostic cue) is the predictor and receiver’s helpfulness is the moderator. | | | | | | |

**Appendix B**

**Further analysis**

**Adding sender’s character traits (continuous)**

In our extended analysis, we also included two covariates: the sender’s own competence and helpfulness, both of which were established during the pre-test. Below, in Table B1 we report the full table of results for the converging (reduced) models that excluded correlations between random effects from the model and the random effects for the interaction term from the model specifications. The table below summarizes the results separately for Experiments 1, 2, and 3.

The regression equation for the final model of Experiments 1 and 3 were the same:

*Sender’s Investment Amount_ij_ = β_0_ + β_1_ Receiver's Helpfulness_ij_ + β_2_Receiver's Competence_ij_ + β_3_ Sender’s Own Helpfulness_ij_ + β_4_ Sender’s Own Competence_ij_ +β_5_(Receiver's Helpfulness_ij_ Receiver's Competence_ij_) + u_0j_ + u_1j_ Receiver's Helpfulness_ij_ + u_2j_Receiver's Competence_ij_ + ε_ij_*^[[3]](#footnote-3)^.

The regression equation for the final model of Experiment 2 was: *Sender’s Investment Amount_ij_ = β_0_ + β_1_ Receiver's Helpfulness_ij_ + β_2_Receiver's Competence_ij_ + β_3_ Sender’s Own Helpfulness_ij_ + β_4_ Sender’s Own Competence_ij_ +β_5_(Receiver's Helpfulness_ij_ Receiver's Competence_ij_) + u_0j_ + u_1j_ Receiver's Helpfulness_ij_ + ε_ij_*^[[4]](#footnote-4)^.

| **Table B1.**  Results of further analysis including sender’s character traits for all three experiments | | | | |
| --- | --- | --- | --- | --- |
| **Mixed Model Anova Table (Type 3 tests, Satterthwaite-method)** | **df** | **F** | ***_η_^2^_p_*** | **p** |
| *Experiment 1* | | | | |
| Receiver’s Helpfulness Reputation | 2, 100 | 77.24 | .61 | <.001 |
| Receiver’s Competence Reputation | 2, 102 | 25.61 | .33 | <.001 |
| Sender’s Competence | 1, 97 | 7.37 | .07 | .008 |
| Sender’s Helpfulness | 1, 97 | 21.19 | .18 | <.001 |
| Receiver’s Helpfulness Reputation * Receiver’s Competence Reputation | 4, 1317 | 6.86 | .02 | <.001 |
| *Experiment 2* | | | | |
| Receiver’s Helpfulness Reputation | 2, 254 | 214.63 | .63 | <.001 |
| Receiver’s Competence Reputation | 2, 3779 | 143.19 | .07 | <.001 |
| Sender’s Competence | 1, 249 | .01 | .00 | .913 |
| Sender’s Helpfulness | 1, 249 | 4.63 | .02 | .032 |
| Receiver’s Helpfulness Reputation * Receiver’s Competence Reputation | 4, 3779 | 16.27 | .02 | <.001 |
| *Experiment 3* | | | | |
| Receiver’s Helpfulness Reputation | 2, 262 | 103.34 | .44 | <.001 |
| Receiver’s Competence Reputation | 2, 256 | 335.30 | .72 | <.001 |
| Sender’s Competence | 1, 251 | 5.77 | .02 | .017 |
| Sender’s Helpfulness | 1, 251 | 4.99 | .02 | .026 |
| Receiver’s Helpfulness Reputation * Receiver’s Competence Reputation | 4, 3289 | 32.35 | .04 | <.001 |
| *Note.* Results of the further (reduced) mixed model, which extended the main models reported in the manuscript by also including covariates for the sender’s competence and helpfulness for each of the three experiments. | | | | |

**Adding sender’s character traits (categorial)**

In this extended analysis, we included two covariates. These were the sender’s own competence and own helpfulness, both of which were established during the pre-test, as categorical variables. We classified each of the sender's character traits into ranking either low, medium or high. However, our cutoffs for classifying senders differed from those of the receivers. To define senders as low, medium or high helpful individuals - across all three experiments - we divided senders by whether their own level of helpfulness was in the bottom, middle or highest third group of all other senders.

Classifying senders' own competence in Experiments 1 and 3 mirrored these cutoffs. In other words, we classified players by whether the number of matrices they solved were in the lowest third, highest third or the middle third. However, for Experiment 2 where competence was operationalized through SAT scores, the cutoffs were defined by the 2023 nationally representative percentiles. Specifically, senders were classified as exhibiting "low" competence if they scored less than 920 points on their SATs and "high" if they scored above 1100 points; those two scored between 921 and 1100 were classified as "medium" competent. Notably, since some senders chose not to answer or did not take the SATs we dropped them from our analysis of Experiment 2, leading to a final sample of *N* = 134.

We report the full table of results for the converging (reduced) models that excluded correlations between random effects from the model and the random effects for the interaction term from the model specifications. That is, the regression equation for the model we report here for all three experiments is: *Sender’s Investment Amount_ij_ = β_0_ + β_1_ Receiver's Helpfulness_ij_ + β_2_Receiver's Competence_ij_ +β_3_(Receiver's Helpfulness_ij_ Receiver's Competence_ij_) + β_4_ Sender’s Own Competence_ij_ +β_5_ Sender’s Own Helpfulness_ij_ +β_6_(Sender’s Own Helpfulness_ij_ Sender’s Own Competence_ij_) +β_7_(Sender’s Own Competence_ij_ Receiver’s Competence_ij_) +β_8_(Sender’s Own Helpfulness_ij_ Receiver’s Helpfulness_ij_)+ u_0j_ + u_1j_ Receiver's Helpfulness_ij_ + ε_ij_*. Table B2 summarizes the results separately for Experiments 1, 2, and 3.

| **Table B2.**  Results of the further (reduced) mixed models for all three experiments | | | | |
| --- | --- | --- | --- | --- |
| **Mixed Model Anova Table (Type 3 tests, Satterthwaite-method)** | **df** | **F** | **η^2^_p_** | **p** |
| *Experiment 1*  Receiver’s Helpfulness Reputation | 2, 98 | 22.56 | .31 | <.001 |
| Receiver’s Competence Reputation | 2, 100 | 25.73 | .34 | <.001 |
| Sender’s Competence | 2, 95 | 3.32 | .07 | .040 |
| Sender’s Helpfulness | 2, 95 | 5.74 | .11 | .004 |
| Receiver’s Helpfulness Reputation * Receiver’s Competence Reputation | 4, 1318 | 6.86 | .02 | <.001 |
| Receiver’s Competence Reputation * Sender’s Competence Reputation | 4, 100 | 0.87 | .03 | .487 |
| Receiver’s Helpfulness Reputation * Sender’s Helpfulness Reputation | 4, 98 | 1.08 | .04 | .370 |
| *Experiment 2*  Receiver’s Helpfulness Reputation | 2, 133 | 138.65 | .68 | <.001 |
| Receiver’s Competence Reputation | 2, 2007 | 18.36 | .02 | <.001 |
| Sender’s Own Competence | 2, 129 | 1.54 | .02 | .218 |
| Sender’s Own Helpfulness | 2, 129 | 1.50 | .02 | .228 |
| Receiver’s Helpfulness Reputation * Receiver’s Competence Reputation | 4,2007 | 8.02 | .02 | <.001 |
| Receiver’s Competence Reputation * Sender’s Own Competence Reputation | 4, 2007 | 1.18 | .00 | .319 |
| Receiver’s Helpfulness Reputation * Sender’s Own Helpfulness Reputation | 4, 133 | .77 | .02 | .548 |
| *Experiment 3*  Receiver’s Helpfulness Reputation | 2, 260 | 111.72 | .46 | <.001 |
| Receiver’s Competence Reputation | 2, 254 | 380.08 | .75 | <.001 |
| Sender’s Own Competence | 2, 249 | .26 | .00 | .774 |
| Sender’s Own Helpfulness | 2, 249 | 2.92 | .02 | .056 |
| Receiver’s Helpfulness Reputation * Receiver’s Competence Reputation | 4, 3280 | 32.29 | .04 | <.001 |
| Receiver’s Competence Reputation * Sender’s Own Competence Reputation | 4, 254 | 7.63 | .11 | <.001 |
| Receiver’s Helpfulness Reputation * Sender’s Own Helpfulness Reputation | 4, 260 | 5.13 | .07 | <.001 |
| *Note.* Results of the further (reduced) mixed model, which extended the main models reported in the manuscript by also including covariates for the sender’s own competence and sender’s own helpfulness for each of the three experiments. | | | | |

Across all three experiments we found significant main effects of both of the receivers' character traits on senders' investment behavior. However, only in Experiment 1 did the senders' own competence and own helpfulness significantly influence their investment behavior. In Experiments 2 and 3 these covariates were not significant predictors. Further, across all three experiments there was a significant interaction effect between the receivers' competence and helpfulness reputation. However, in Experiments 1 and 2 there was neither a significant interaction between senders' own helpfulness and receivers' helpfulness nor an interaction between senders' own competence and receivers' competence. Only Experiment 3 showed a significant interaction effect between, both, the senders' own and receivers' competence and their helpfulness (see Table B2). To explore these interaction effects further, we conducted a post-hoc follow-up pairwise comparison of these effects.

In Table B3 we report the marginal means and in Table B4 we provide an overview of the pairwise comparison of senders' own helpfulness and receivers' helpfulness. We found that for receivers with a low and medium reputation for helpfulness, senders' own helpfulness did not significantly influence their investing behavior. However, when receivers had a high reputation for helpfulness, highly helpful senders also tended to invest significantly more compared to senders ranking medium or low on helpfulness.

In Table B5 we report the marginal means and in Table B6 we provide an overview of the pairwise comparison of senders' own competence and receivers' competence. We find that senders who ranked low on their own competence tended to invest significantly more to low competent receivers, compared to highly competent receivers. We do not find that the investment to low competent receivers significantly differs between low and medium or high and medium senders. Receivers who have a medium reputation for competence, are not met with significantly different investments by low, medium, or high competent senders. However, while we do find a positive effect between senders' own competence level and their level of investments towards highly competent receivers - the difference is not always significant, as revealed by the pairwise comparison.

| **Table B3.**  Experiment 3 marginal means by senders’ own helpfulness and receivers’ helpfulness level | | | | | |
| --- | --- | --- | --- | --- | --- |
| **Receiver’s**  **Helpfulness**  **Reputation** | **Sender’s Own**  **Helpfulness** | **emmeans** | **SE** | **df** | **CI** |
| Low | High | 28.74 | 1.77 | 336 | [25.26, 32.23] |
|  | Medium | 24.94 | 1.84 | 334 | [21.33, 28.55] |
|  | Low | 28.52 | 1.72 | 336 | [25.14, 31.90] |
| Medium | High | 36.28 | 1.68 | 277 | [32.98, 39.58] |
|  | Medium | 31.27 | 1.74 | 276 | [27.85, 34.70] |
|  | Low | 33.26 | 1.63 | 277 | [30.06, 36.46] |
| High | High | 43.96 | 1.77 | 336 | [40.47, 47.44] |
|  | Medium | 36.34 | 1.84 | 334 | [32.72, 39.95] |
|  | Low | 35.83 | 1.72 | 336 | [32.74, 39.94] |
| Dependent Variable: Senders’ Level of Investment  Degrees-of-freedom method: Kenward-Roger  Confidence level: 0.95 | | | | | |

| **Table B4.**  Experiment 3 results for follow-up pairwise comparison tests, comparing the receivers’ helpfulness and senders’ own helpfulness on the sender’s investment choices | | | | | | |
| --- | --- | --- | --- | --- | --- | --- |
| **Receiver’s**  **Helpfulness**  **Reputation** | **Sender’s Own**  **Helpfulness** | **Estimate** | **SE** | **df** | **t** | **p** |
| Low | High - Low | .22 | 2.47 | 336 | .09 | 1.000 |
|  | High - Medium | 3.80 | 2.53 | 337 | 1.50 | .404 |
|  | Low - Medium | 3.58 | 2.51 | 335 | 1.43 | .465 |
| Medium | High - Low | 3.02 | 2.34 | 277 | 1.29 | .592 |
|  | High - Medium | 5.01 | 2.40 | 277 | 2.09 | .114 |
|  | Low - Medium | 1.98 | 2.38 | 277 | 0.83 | 1.000 |
| High | High - Low | 8.13 | 2.47 | 336 | 3.29 | .003 |
|  | High - Medium | 7.62 | 2.53 | 336 | 3.01 | .009 |
|  | Low - Medium | -.50 | 2.51 | 335 | -0.20 | 1.000 |
| Dependent Variable: Senders’ Level of Investment  Degrees-of-freedom method: Kenward-Roger  P value adjustment: Bonferroni method for 3 tests | | | | | | |

| **Table B5.**  Experiment 3 marginal means by sender’s own competence and receiver’s competence level | | | | | |
| --- | --- | --- | --- | --- | --- |
| **Receiver’s**  **Competence**  **Reputation** | **Sender’s Own**  **Competence** | **emmeans** | **SE** | **df** | **CI** |
| Low | High | 14.08 | 2.07 | 445 | [10.00, 18.15] |
|  | Medium | 14.17 | 2.17 | 447 | [9.90, 18.45] |
|  | Low | 20.06 | 1.77 | 447 | [16.59, 23.53] |
| Medium | High | 30.91 | 1.84 | 326 | [27.30, 34.53] |
|  | Medium | 30.20 | 1.92 | 327 | [26.42, 33.98] |
|  | Low | 30.66 | 1.56 | 327 | [27.58, 33.73] |
| High | High | 57.64 | 2.14 | 499 | [53.43, 61.86] |
|  | Medium | 54.01 | 2.25 | 503 | [49.59, 58.43] |
|  | Low | 47.42 | 1.83 | 503 | [43.83, 51.01] |
| Dependent Variable: Senders’ Level of Investment  Degrees-of-freedom method: Kenward-Roger  Confidence level: 0.95 | | | | | |

| **Table B6.**  Experiment 3 results for follow-up pairwise comparison tests, comparing the receivers’ competence and senders’ own competence on the sender’s investment choices | | | | | | |
| --- | --- | --- | --- | --- | --- | --- |
| **Receiver’s**  **Competence**  **Reputation** | **Sender’s Own**  **Competence** | **Estimate** | **SE** | **df** | **t** | **p** |
| Low | High - Low | -5.98 | 2.73 | 445 | -2.19 | .087 |
|  | High - Medium | -.10 | 3.01 | 446 | -.03 | 1.000 |
|  | Low - Medium | 5.89 | 2.80 | 447 | 2.10 | .109 |
| Medium | High - Low | .26 | 2.42 | 326 | .11 | 1.000 |
|  | High - Medium | .72 | 2.66 | 326 | .27 | 1.000 |
|  | Low - Medium | .46 | 2.48 | 327 | .19 | 1.000 |
| High | High - Low | 10.23 | 2.83 | 499 | 3.62 | .001 |
|  | High - Medium | 3.64 | 3.11 | 501 | 1.17 | .728 |
|  | Low - Medium | -6.59 | 2.90 | 502 | -2.27 | .071 |
| Dependent Variable: Senders’ Level of Investment  Degrees-of-freedom method: Kenward-Roger  Confidence level: 0.95 | | | | | | |

***Examining the role of senders’ own characteristic traits***

For each experiment, we calculated the marginal means of the main effects of each of the senders' own character traits. That is - aggregating over senders' own competence - for each sender whose own helpfulness ranked low, medium, or high, we calculated the marginal means of their investment behavior (Table B7) followed by pairwise comparisons (Table B8). Further, aggregating over senders' own helpfulness, we calculated the marginal means of the investments senders' whose own competence was ranked low, medium, and high (Table B9) and pairwise comparisons (Table B10). Finally, we calculate the correlation between senders' own competence and helpfulness.

**Sender's helpfulness.** In Experiments 1 and 2, where the outcome-relevant characteristic was helpfulness, we observed that senders tended to invest more with receivers who were perceived as more helpful (see Table B7). However, post-hoc pairwise comparisons showed that the differences in Experiment 2 were not statistically significant; in Experiment 1, significant differences were found between senders who ranked *High* vs. *Low* and between those who ranked *Low* and *Medium* in terms of their helpfulness, but not between the *High* and *Medium* conditions (see Table B8).

In Experiment 3, where helpfulness was not the diagnostic characteristic, highly helpful senders tended to invest the most; senders ranking as the lowest in helpfulness invested more than those ranked as medium helpers (see Table B7). However, our post-hoc pairwise comparison revealed that the only significant difference was between senders who ranked high and medium in terms of their helpfulness. The differences in investments between high - low, and low - medium helpful senders were not significant (see Table B8).

Hence, we can conclude that when the outcome-relevant cue is helpfulness (Experiments 1 and 2), there was a positive relationship between senders' helpfulness and the size of their investment - though the difference was not always significant relative to players with medium or low helpfulness levels.

| **Table B7.**  Marginal means of senders’ own helpfulness for all three experiments | | | | |
| --- | --- | --- | --- | --- |
| **Marginal Means of Sender’s Own Helpfulness** | **emmeans** | **SE** | **df** | **CI** |
| *Experiment 1*  High | 29.68 | 4.71 | 95 | [20.32, 39.04] |
| Medium | 21.83 | 1.19 | 95 | [19.48, 24.19] |
| Low | 16.54 | 1.57 | 95 | [13.42, 19.66] |
| *Experiment 2*  High | 24.55 | 2.55 | 129 | [19.50, 29.60] |
| Medium | 22.39 | 2.75 | 129 | [16.95, 27.84] |
| Low | 20.49 | 2.49 | 129 | [15.56, 25.42] |
| *Experiment 3*  High | 36.33 | 1.63 | 249 | [33.11, 39.54] |
| Medium | 30.85 | 1.70 | 249 | [27.51, 34.19] |
| Low | 32.54 | 1.58 | 249 | [29.42, 35.65] |
| Degrees-of-freedom method: Kenward-Roger  Confidence level: 0.95  *Note.* Initial endowments in Experiments 1 and 2 were 50 pence, in Experiment 3 100 pence. Sender’s own helpfulness classifications: based on equally divided data into three groups | | | | |

| **Table B8.**  Sender’s own helpfulness follow-up pairwise comparison tests, for all three experiments | | | | | | | |
| --- | --- | --- | --- | --- | --- | --- | --- |
|  | **Contrast** | **Estimate** | **SE** | **df** | **t** | **p** |  |
| *Experiment 1* | High - Low | 13.14 | 4.95 | 95 | 2.66 | .028 |  |
|  | High - Medium | 7.85 | 4.86 | 95 | 1.61 | .329 |  |
|  | Low - Medium | -5.29 | 1.97 | 95 | -2.69 | .026 |  |
| *Experiment 2* | High - Low | 4.06 | 2.35 | 129 | 1.73 | .259 |  |
|  | High - Medium | 2.15 | 2.61 | 129 | .83 | 1.000 |  |
|  | Low - Medium | -1.90 | 2.47 | 129 | -0.77 | 1.000 |  |
| *Experiment 3* | High - Low | 3.79 | 2.28 | 249 | 1.67 | .291 |  |
|  | High - Medium | 5.48 | 2.33 | 249 | 2.35 | .059 |  |
|  | Low - Medium | 1.69 | 2.32 | 249 | .73 | 1.000 |  |
| Dependent Variable: Sender’s own level of investment  Degrees-of-freedom method: Kenward-Roger  P value adjustment: Bonferroni method for 3 tests | | | | | | |  |

**Sender's competence**. We found no clear, linear relationship between senders' own competence and their investment behavior. That is, across all three experiments, there was no indication that more competent senders invested significantly more than less competent senders.

In Experiments 1 and 3, where senders' own competence was defined by the relative number of matching games they correctly solved, highly competent senders generally chose to invest the largest portion of their endowment (see Table B9). However, the size of this investment was not significantly different from that made by senders whose competence rank was low or medium (see Table B10). In Experiment 2, low competent senders tended to make the highest investments (see Table B9); however, this was also not significantly different from the investments made by senders with other competence levels (see Table B10). Additionally, it should be noted that the results of Experiment 2 are based on a reduced dataset (*N* = 139), excluding senders who chose not to provide their SAT scores; this may have introduced bias due to the exclusion of many low competent senders who may have been less willing to provide their SAT scores than if they ranked high.

Hence, we can conclude that across all three experiments senders who were more competent did not generally choose to invest more than less competent senders.

| **Table B9.**  Sender’s own helpfulness follow-up pairwise comparison tests, for all three experiments | | | | |
| --- | --- | --- | --- | --- |
| **Marginal Means of Sender’s Own Competence** | **emmeans** | **SE** | **df** | **CI** |
| *Experiment 1*  High | 26.17 | 2.33 | 95 | [21.55, 30.78] |
| Medium | 20.96 | 2.21 | 95 | [16.57, 25.35] |
| Low | 20.93 | 1.94 | 95 | [17.07, 24.78] |
| *Experiment 2*  High | 21.43 | 1.06 | 129 | [19.33, 23.53] |
| Medium | 17.70 | 4.72 | 129 | [8.36 27.03] |
| Low | 28.31 | 4.36 | 129 | [19.69, 36.92] |
| *Experiment 3*  High | 34.21 | 1.71 | 249 | [30.85, 37.57] |
| Medium | 32.79 | 1.78 | 249 | [29.28, 36.31] |
| Low | 32.71 | 1.45 | 249 | [29.86, 35.57] |
| Degrees-of-freedom method: Kenward-Roger  Confidence level: 0.95  *Note.* Initial endowments in Experiments 1 and 2 were 50 pence, in Experiment 3 100 pence. Competence classifications in Experiments 1 and 3 were based on equally divided data into three groups. In Experiment 2 competence was based on the 33% and 66% cutoff of nationally reported SAT scores in 2023: participants who reported a score under 920 were ranked low, above 1100 ranked high and between the two ranked medium. | | | | |

| **Table B10.**  Sender’s own competence follow-up pairwise comparison tests, for all three experiments | | | | | | |
| --- | --- | --- | --- | --- | --- | --- |
|  | **Contrast** | **Estimate** | **SE** | **df** | **t** | **p** |
| *Experiment 1* | High - Low | 5.24 | 2.28 | 95 | 2.30 | .071 |
|  | High - Medium | 5.21 | 2.36 | 95 | 2.21 | .089 |
|  | Low - Medium | -.03 | 2.26 | 95 | -.01 | 1.000 |
| *Experiment 2* | High - Low | -6.88 | 4.47 | 129 | -1.54 | .380 |
|  | High - Medium | 3.73 | 4.83 | 129 | 0.77 | 1.000 |
|  | Low - Medium | 10.61 | 6.42 | 129 | 1.65 | .302 |
| *Experiment 3* | High - Low | 1.50 | 2.25 | 249 | .67 | 1.000 |
|  | High - Medium | 1.42 | 2.47 | 249 | .57 | 1.000 |
|  | Low - Medium | -.08 | 2.30 | 249 | -.04 | 1.000 |
| Dependent Variable: Sender’s own level of investment  Degrees-of-freedom method: Kenward-Roger  P value adjustment: Bonferroni method for 3 tests | | | | | | |

**Correlation between senders' own competence and own helpfulness.** We analyzed the zero-order relationship between senders' own competence and senders' own helpfulness. As shown in Figure 5, and confirmed through a Shapiro-Wilk test, both of the senders' character traits were not normally distributed. Therefore, we calculated Spearman's correlation coefficient between the senders' two character traits for each experiment.

In Experiment 1, where the outcome-relevant cue was helpfulness the correlation between senders' two character traits was close to zero and not statistically significant (ρ = -0.02, *p* = .439). In both Experiments 2 and 3, where the informative reputation cue was the receivers' competence, our analysis showed a small but significant negative correlation between senders' competence and their helpfulness (Exp. 2: ρ= -0.20 *p* < .001; Exp. 3: ρ = -0.11, *p* < .001). Hence, in these experiments, more competent senders were slightly less helpful.

1. β_0_ is the intercept, β_1_, β_2_, β_3_ are the fixed effects coefficients; *u_0j_* is the random intercept and *u_1j_* and *u_2j_* are the random slopes for the receiver *j;* *ε_ij_* is the residual error term. [↑](#footnote-ref-1)
2. β_0_ is the intercept, β_1_, β_2_, β_3_ are the fixed effects coefficients; *u_0j_* is the random intercept and *u_1j_* are the random slopes for the helpfulness reputation within each receiver *j;* *ε_ij_* is the residual error term. [↑](#footnote-ref-2)
3. β_0_ is the intercept, β_1_, β_2_, β_3_, β_4_, β_5_ are the fixed effects coefficients; *u_0j_* is the random intercept and *u_1j_* and *u_2j_* are the random slopes for the receiver *j’s* competence and helpfulness reputation*;* *ε_ij_* is the residual error term. [↑](#footnote-ref-3)
4. β_0_ is the intercept, β_1_, β_2_, β_3_ are the fixed effects coefficients; *u_0j_* is the random intercept and *u_1j_* are the random slopes for the helpfulness reputation of receiver *j;* *ε_ij_* is the residual error term. [↑](#footnote-ref-4)
